# cGMP dynamics that underlies thermosensation in temperature-sensing neuron regulates thermotaxis behavior in *C*. *elegans*

**DOI:** 10.1371/journal.pone.0278343

**Published:** 2022-12-06

**Authors:** Ichiro Aoki, Makoto Shiota, Yuki Tsukada, Shunji Nakano, Ikue Mori

**Affiliations:** 1 Group of Molecular Neurobiology, Neuroscience Institute, Graduate School of Science, Nagoya University, Nagoya, Japan; 2 Department of Biological Science, Graduate School of Science, Nagoya University, Nagoya, Japan; University of Kansas College of Liberal Arts and Sciences, UNITED STATES

## Abstract

Living organisms including bacteria, plants and animals sense ambient temperature so that they can avoid noxious temperature or adapt to new environmental temperature. A nematode *C*. *elegans* can sense innocuous temperature, and navigate themselves towards memorize past cultivation temperature (T_c_) of their preference. For this thermotaxis, AFD thermosensory neuron is pivotal, which stereotypically responds to warming by increasing intracellular Ca^2+^ level in a manner dependent on the remembered past T_c_. We aimed to reveal how AFD encodes the information of temperature into neural activities. cGMP synthesis in AFD is crucial for thermosensation in AFD and thermotaxis behavior. Here we characterized the dynamic change of cGMP level in AFD by imaging animals expressing a fluorescence resonance energy transfer (FRET)-based cGMP probe specifically in AFD and found that cGMP dynamically responded to both warming and cooling in a manner dependent on past T_c_. Moreover, we characterized mutant animals that lack guanylyl cyclases (GCYs) or phosphodiesterases (PDEs), which synthesize and hydrolyze cGMP, respectively, and uncovered how GCYs and PDEs contribute to cGMP and Ca^2+^ dynamics in AFD and to thermotaxis behavior.

## Introduction

Thermosensation is universal from bacteria to plants and animals, and enables organisms to react to noxious temperature or adapt to new environmental temperature [1, 2]. *C*. *elegans* senses not only noxiously high and low temperature [3–5] but also innocuous temperature, which was indicated by thermotaxis behavior, where animals cultivated at certain temperature migrate toward that temperature on a thermal gradient [6]. Thermotaxis indicates that *C*. *elegans* can sense temperature, memorize past cultivation temperature (T_c_), and compare the present temperature with the memorized past temperature. AFD is a major thermosensory neuron responsible for thermotaxis of *C*. *elegans* [7, 8], which responds to warming by increasing its intracellular Ca^2+^ level in a manner dependent on past T_c_ [9–14]. This property of AFD is conserved even when AFD is disconnected from neural circuits in culture *in vitro* [15], indicating that AFD can cell-autonomously sense temperature, memorize past T_c_ and compare the present temperature with the memorized past temperature.

For AFD thermosensation, three guanylyl cyclases (GCYs), GCY-8, GCY-18 and GCY-23, which are specifically expressed in AFD, and cyclic nucleotide-gated (CNG) channels TAX-4 and TAX-2, which are far more sensitive to cGMP than to cAMP, are essential [15–19]: AFD in animals lacking these three GCYs, TAX-4 or TAX-2 does not respond to warming. Ectopic expression of these GCYs are sufficient to confer thermo-responsiveness to chemosensory neurons, which are otherwise irresponsive to temperature change, suggesting a possibility that these GCYs are temperature sensors [20]. One GCY is known to function in thermosensation as well in mammals; the subtype G of transmembrane guanylyl cyclase (GC-G) in the Grueneberg ganglion (GG) in the mouse nose is activated by cool temperature and necessary for cold-evoked behavior of mice [21]. GC-G activates a cGMP-activated channel, CNGA3, which is proposed to mediate cool-evoked response of GG neurons [21–23]. Thus, thermosensation by transmembrane guanylyl cyclases and subsequent activation of CNG channels might be a conserved mechanism of thermosensory transduction in different phyla.

Genetic experiments indicated that cGMP should transduce temperature information to Ca2+ dynamics in AFD neurons of *C*. *elegans*. It was indeed reported recently that cGMP level in AFD increases in response to warming in a manner dependent on past T_c_ similarly to Ca^2+^ dynamics [24]. We further investigated cGMP dynamics in AFD by expressing a genetically encoded FRET probe for cGMP, cGi-500 [25] and by applying more complex temperature programs. FRET probes like cGI-500 are ratio-metric and cancel out difference in expression level of the probes in different strains if any. We found that cGMP level increased and decreased in response to warming and cooling, respectively, specifically at a sensory ending of AFD in a manner dependent on past T_c_, which is consistent with the previous report [24].

The three GCYs, GCY-8, GCY-18 and GCY-23, expressed in AFD redundantly but differentially contribute to thermotaxis and thermosensation of AFD. Three of *gcy* double mutants, in which only one *gcy* gene out of the three is intact, exhibit differential abnormality in thermotaxis behavior [16] and AFD Ca2+ dynamics [11, 20]. GCY-23 and GCY-18 seems to have more important roles in thermosensation at lower and higher temperature range, respectively, since when GCY-23 and GCY-18 are ectopically expressed in chemosensory neurons, they confer low and high onset temperature regardless of animals’ T_c_, respectively [20]. We therefore examined how each GCY contributes to cGMP dynamics in AFD by imaging *gcy* double mutants and uncovered differential contributions of these GCYs to cGMP dynamics in AFD.

cGMP is hydrolyzed by phophodiesterases (PDEs). Of six PDEs in *C*. *elegans*, four PDEs (PDE-1, PDE-2, PDE-3 and PDE-5) are supposed to hydrolyze both cGMP and cAMP, whereas PDE-4 and PDE-6 are supposed to be cAMP-specific [26–28]. These PDEs capable of cGMP hydrolysis are involved in phototransduction in ASJ photosensory neurons [28] and thermotaxis [29]. We investigated how these PDEs affect spatio-temporal dynamics of cGMP and Ca^2+^ in AFD and uncovered complex co-operation among the PDEs to properly form AFD Ca^2+^ dynamics and thermotaxis behavior.

## Results

### cGMP level in AFD dynamically responds to temperature change

**Fig 1:**
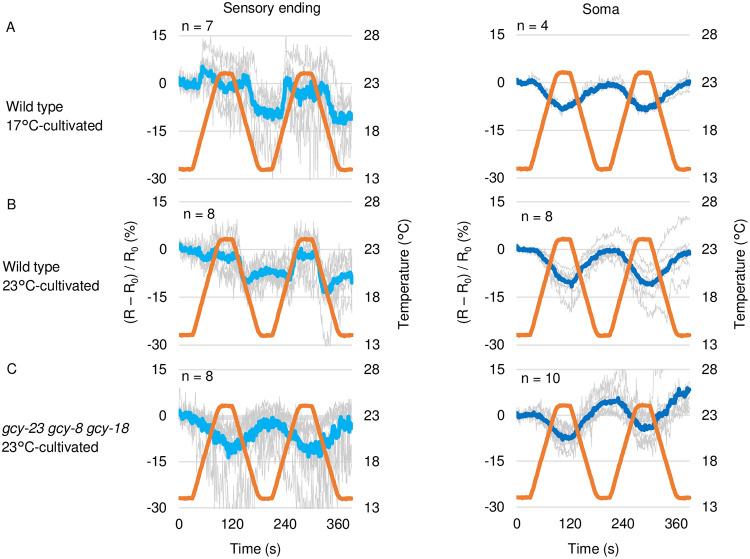
cGMP dynamics in AFD. A and B. Wild type animals expressing cGi-500 cGMP indicator specifically in AFD thermosensory neurons (IK3110) were cultivated at 17°C (A) or 23°C (B). Blue and yellow fluorescence was monitored during warming from 14°C to 23°C and subsequent cooling to 14°C, which were repeated twice as indicated (orange line). Warming and cooling was at the rate of 1°C/6 sec. Individual (gray) and average fluorescence ratio (CFP/YFP) change at AFD sensory ending (blue) and soma (dark blue) is shown. The temperature program was repeated twice since the increment of the fluorescence ratio was more remarkable in response to the second warming especially in 23°C-cultivate animals, probably due to the fluorescence ratio was once decreased by the first round of cooling. C. *gcy-18 gcy-8 gcy-23* triple mutant animals expressing cGi-500 cGMP indicator in AFD (IK3360) were cultivated at 23°C and subjected to imaging analysis. R_0_ is average of R (CFP/YFP) from t = 1 to t = 30.

To reveal whether and how cGMP level changes in AFD, we expressed a genetically encoded FRET-based cGMP probe, cGi-500, which increases CFP/YFP fluorescence ratio when cGMP concentration increases [25], specifically in AFD and performed imaging analyses. CFP/YFP fluorescence ratio at the AFD sensory ending of wild type animals expressing cGi-500 cultivated at 17°C or 23°C increased in response to warming in a manner dependent on the past T_c_ as described previously using FlincG, a GFP-based cGMP probe [24]. Our result is in consistent with previous description for cGMP [24] and Ca^2+^ [9] during warming. The CFP/YFP ratio decreased in response to cooling also in a manner dependent on the past T_c_ (Fig 1A and 1B, left).

The fluorescence ratio change seen in wild type was not observed in *gcy-23 gcy-8 gcy-18* triple mutant animals (Fig 1C), in which AFD Ca^2+^ dynamics and thermotaxis behavior are abolished [15, 16] and therefore AFD cGMP dynamics are supposed to be abolished. In *gcy-23 gcy-8 gcy-18* triple mutant animals, the CFP/YFP ratio rather anti-correlated with the warming and cooling, respectively (Fig 1C), which is considered to be derived from the direct effect of temperature change on the probe or the focus. Given the opposite trend in the increment and decrement of CFP/YFP ratio between wild type and *gcy-23 gcy-8 gcy-18* triple mutant animals (Fig 1B and 1C), the CFP/YFP ratio change observed at the sensory endings of wild type animals is likely to reflect the genuine change of cGMP level.

*gcy-8 gcy-18 gcy-23* mutants show abnormal morphology of AFD sensory ending lacking microvilli [30], from which we imaged. To decouple the effects of the structural abnormality and the loss of cGMP synthesis in the *gcy-8 gcy-18 gcy-23* mutants, we attempted to image in *tax-4* mutants. After transferring the extrachromosomal array expressing CGI-500 to *tax-4* mutant, however, the CGI-500 probe was totally invisible. There might be feedback from Ca^2+^ influx through the CNG channels to transcription from the *gcy-8* promoter.

Since cGMP activates TAX-2/TAX-4 cGMP-gated cation channel [17, 19, 20] thereby resulting in Ca^2+^ influx, it had been possible that the detection of temperature change and the comparison between the past and present temperature could be done anywhere between GCYs and Ca^2+^ influx and reflected on Ca^2+^ dynamics. Our and another group’s [24] results indicate that these processing of temperature information is already reflected on the cGMP dynamics.

In AFD soma, CFP/YFP fluorescence ratio seemed to decrease and increase in an anti-correlating manner to warming and cooling, respectively (Fig 1, right), similarly to the AFD sensory ending in *gcy-23 gcy-8 gcy-18* triple mutants. These results support that the cGMP level changes specifically at the sensory ending in AFD as described previously [24], and are consistent with the sensory ending-specific localization of GCYs [16, 31] and the result that the laser- axotomized AFD sensory ending but not AFD soma changes Ca^2+^ level in response to temperature stimuli [12]. Compartmentalized cGMP dynamics were observed also in other sensory neurons in *C*. *elegans* such as chemosensory AWC [32] and O2-sensing PQR [33], suggesting that the cGMP level is commonly regulated in a spatially restricted manner possibly by prevention of cGMP diffusion or by catabolism.

### Guanylyl cyclases (GCYs) regulate onset temperature for change of cGMP level in AFD

**Fig 2:**
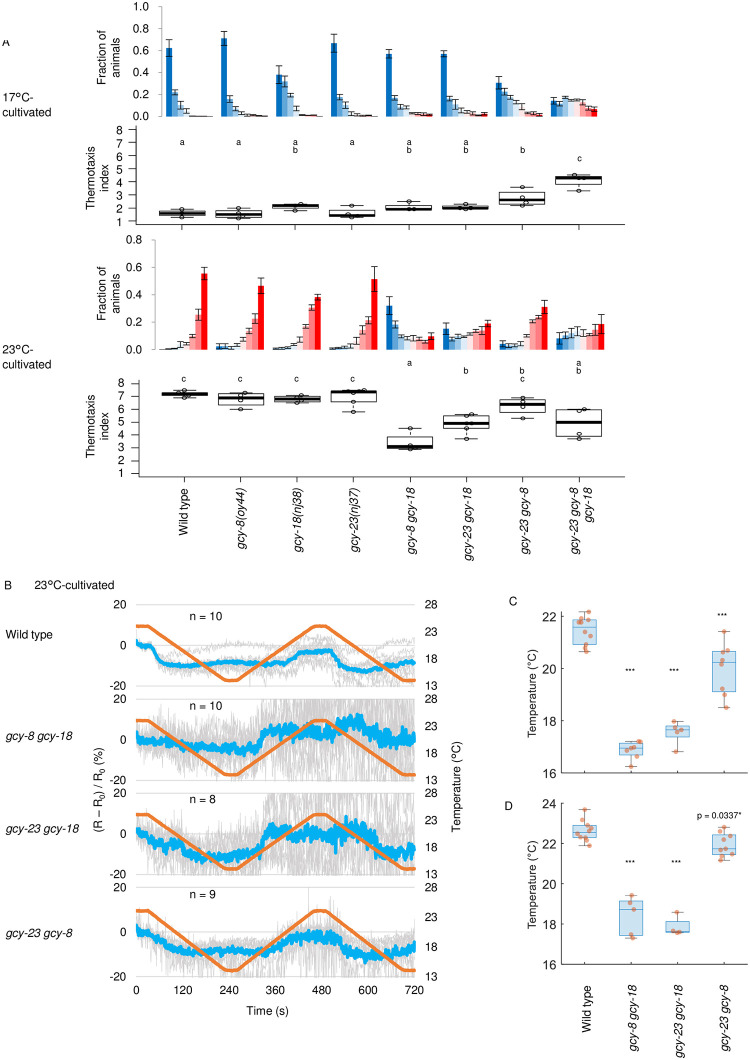
cGMP onsets from lower temperature in *gcy* double mutants. A. Wild type animals and animals in which indicated *gcy* gene(s) is mutated were cultivated at 17°C or 23°C and then placed on a thermal gradient. The number of animals in each section of the thermal gradient was scored, and the proportion of animals in each section was plotted on histograms. n = 3 to 6 as indicated by open circles in boxplots. The error bars in histograms represent the standard error of mean (SEM). The thermotaxis indices were plotted on boxplots. The indices of strains marked with distinct alphabets differ significantly (p < 0.05) according to the Tukey-Kramer test. B. Wild type and indicated *gcy* double mutant animals that express cGi-500 cGMP indicator in AFD were cultivated at 23°C and subjected to imaging analysis with temperature stimuli indicated (orange line). Warming and cooling was at the rate of 1°C/20 sec. Individual (gray) and average (blue) fluorescence ratio (CFP/YFP) change at AFD sensory ending was plotted against time. A temperature program with slower change rate than in Fig 1 was used to compare the onset temperature between different strains. Since the increment of the fluorescence ratio was more remarkable in response to the warming after cooling, the temperature program starting from cooling was used to shorten measurement time and therefore to prevent the probe from bleaching. C and D. Temperature at which cGMP level started increasing in response to warming (C) and decreasing in response to the 2^nd^ cooling (D) was extracted using a MATLAB command ‘findchangepts’ as detailed in S1 Fig and plotted. *** indicates p < 0.001 (Dunnett test against wild type animals).

Three GCYs expressed in AFD, GCY-8, GCY-18 and GCY-23, redundantly but differentially contribute to thermosensation of AFD and thermotaxis. Three of *gcy* double mutants, in which only one *gcy* gene out of the three is intact, exhibited differential abnormality in thermotaxis tested for the populations of animals on a linear gradient (Fig 2A) as we previously reported for thermotaxis tested for individual animals on a radial gradient [16]. The behavioral abnormality was consistent with the suggested roles of GCY-23 and GCY-18 at lower and higher temperature range, respectively [20]; *gcy-8 gcy-18* and *gcy-23 gcy-8* mutants, which carry only GCY-23 and GCY-18, respectively, out of the three, were abnormal when cultivated at 23°C and 17°C, respectively (Fig 2A).

We then examined how each GCY contributes to cGMP dynamics in AFD by imaging *gcy* double mutants cultivated at 23°C, since the abnormality in thermotaxis was clearer when animals were cultivated at 23°C.

AFD in all the three *gcy* double mutant animals cultivated at 23°C exhibited lower onset temperature for cGMP increment and decrement than AFD in wild type animals (Fig 2B–2D). The extent of abnormality was severe in *gcy-8 gcy-18* and *gyc-23 gcy-18* but less in *gcy23 gcy-8* double mutants. These results were well correlated to abnormality in the Ca^2+^ dynamics and the thermotaxis behavior of animals cultivated at 23°C; abnormality of onset temperature for Ca^2+^ increment (S2 & S3 Figs, right) and of thermotaxis (Fig 2A, lower) was severe in *gcy-8 gcy-18* and *gyc-23 gcy-18* but less in *gcy23 gcy-8* double mutants. These results support a model that GCY-23 and GCY-8 are activated at lower temperature than GCY-18, rather than another model that the temperature-specific contribution of each GCY to the Ca^2+^ dynamics is derived from some other temperature-specific processes downstream of GMP synthesis such as provision of cGMP from each GCY to CNG channels. Contribution of the latter possibility is, however, not excluded. Increased noise in cGMP dynamics of *gcy-8 gyc-18* and *gcy-23 gcy-18* double mutants was probably due to low fluorescence intensity caused by low expression of the probe (S4A Fig). Therefore, GCY-18 might positively regulate expression from *gcy-8* promoter.

Given that cGMP increment is suppressed below the threshold in wild type animals with GCY-23 and GCY-8, which are activated at lower temperature when existing alone (Fig 2B), and that *gcy-18* single mutant, in which GCY-23 and GCY-8 also co-exist, show mostly normal thermotaxis (Fig 2A) and Ca^2+^ dynamics [11], GCY-23 and GCY-8 might prevent activation of each other below the threshold. Consistently, basal cGMP seemed increased in *gcy-8 gyc-18* and *gcy-23 gcy-18* double mutants (S4B Fig), again suggesting that GCY-23 and GCY-8 are aberrantly active when existing alone. Taken together, the cGMP dynamics in AFD seems to be composed of differential contribution from GCY-8, GCY-18 and GCY-23, and that Ca^2+^ level immediately increases in response to cGMP increment.

Thermotaxis index of *gcy-8 gcy-18* double mutants cultivated at 23°C was aberrantly lower than that of *gcy-23 gcy-8 gcy-18* triple mutants (Fig 2A), while the cGMP dynamics were more impaired in *gcy-23 gcy-8 gcy-18* triple mutants (Figs 1C and 2B). This is supposed to be because *gcy-23 gcy-8 gcy-18* triple mutant is totally insensitive to temperature [15] and therefore spreads evenly on a thermal gradient [16] (Fig 2A); on the other hand, *gcy-8 gcy-18* double mutants were still able to sense temperature but in an abnormal way, where cGMP and Ca2+ increased in response to warming from much lower temperature than their T_c_ as if the animals had been cultivated at lower temperature (Fig 2B and S2 & S3 Figs). This misrepresentation of temperature information in *gcy-8 gcy-18*, derived from misprocessing of either past T_c_ or current temperature, probably drove the animals toward the cold regions of thermal gradient.

### Phosphodiesterases (PDEs) contribute to proper thermotaxis

**Fig 3:**
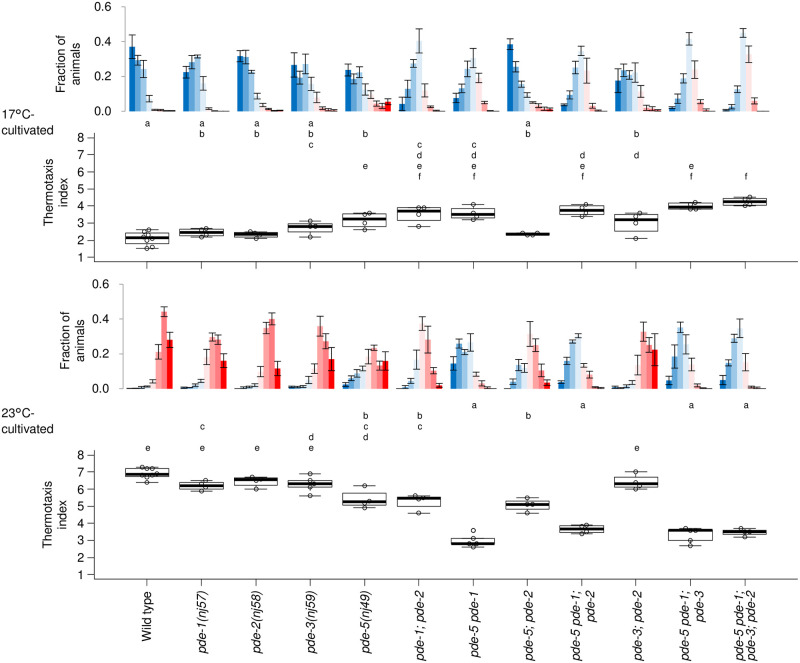
*pde* mutants are defective for thermotaxis behavior. Wild type and *pde* mutant animals indicated were cultivated at 17°C or 23°C and then subjected to thermotaxis assay. n = 4 to 8 as indicated by open circles in boxplots. The error bars in histograms represent the standard error of mean (SEM). The thermotaxis indices of strains marked with distinct alphabets differ significantly (p < 0.05) according to the Tukey-Kramer test.

We next aimed to analyze how PDEs capable of cGMP hydrolysis affect spatio-temporal dynamics of cGMP and Ca^2+^ in AFD and thermotaxis behavior. First, we analyzed thermotaxis of *pde* mutants. Among single mutants for *pde-1*, *pde-2*, *pde-3 and pde-5*, product of which hydrolyze cGMP, *pde-5* mutants were significantly defective in thermotaxis when cultivated at both 17°C and 23°C (Fig 3). Since at least *pde-1*, *pde-2* and *pde-5* are expressed in AFD [29, 34], we then analyzed double and triple mutants for these three genes to examine whether these PDEs redundantly function in AFD. *pde-1; pde-2* double mutants were defective when cultivated at both 17°C and 23°C. Mutations in both *pde-5* and *pde-1* exhibited a synergistic effect especially when cultivated at 23°C. *pde-2* mutation did not enhance abnormality of *pde-5* nor *pde-5 pde-1*. Effect of *pde-3* mutation in addition to other mutations was marginal (Fig 3). None of *pde* mutants showed apparent locomotion defect on NGM plates. These results suggest that PDE-1, PDE-2, and PDE-5 co-operate to regulate thermotaxis.

### *pde-5* acts in AFD to regulate cGMP dynamics and thermotaxis behavior

**Fig 4:**
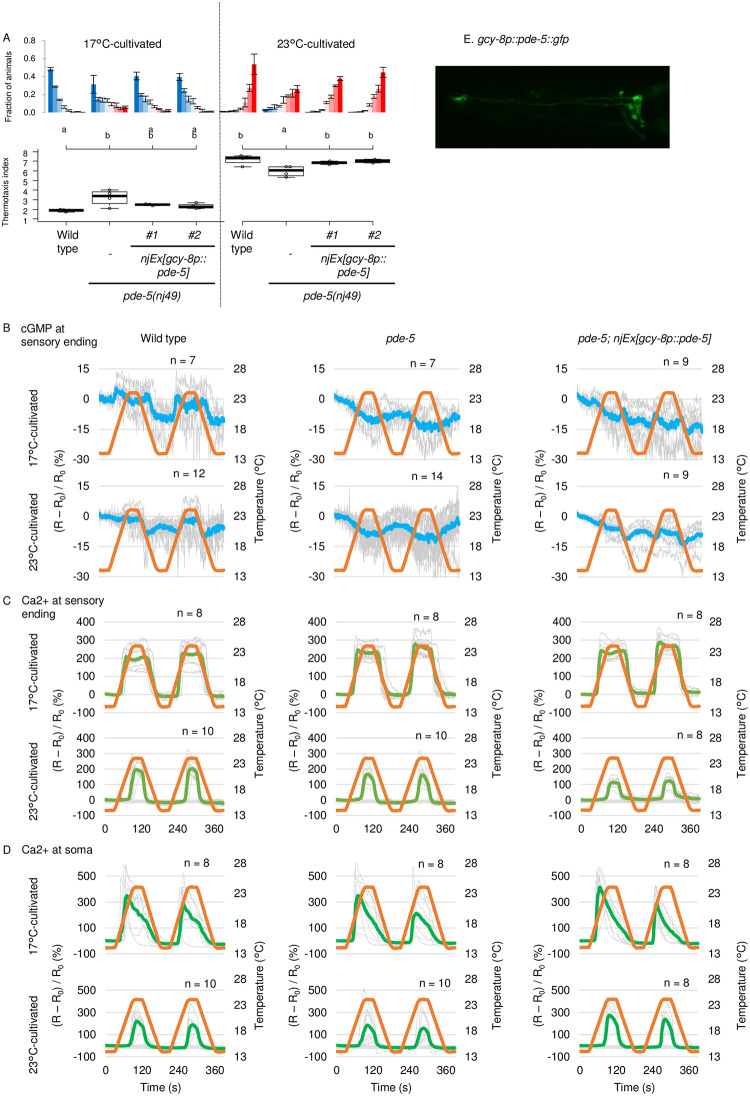
*pde-5* acts in AFD to regulate cGMP dynamics and thermotaxis. A. Wild type and *pde-5* animals and *pde-5* animals that express PDE-5 specifically in AFD were cultivated at 17°C or 23°C and then subjected to thermotaxis assay. n = 4. The error bars in histograms represent the standard error of mean (SEM). The thermotaxis indices of strains marked with distinct alphabets differ significantly (p < 0.05) according to the Tukey-Kramer test. B. Wild type and *pde-5* animals and *pde-5* mutant animals expressing PDE-5 in AFD that express cGi-500 cGMP indicator in AFD were cultivated at 17°C or 23°C. Animals were then subjected to imaging analysis with temperature stimuli indicated (orange line). Warming and cooling was at the rate of 1°C/6 sec. Individual (gray) and average (blue) fluorescence ratio (CFP/YFP) change at AFD sensory ending is shown. Dataset of wild type cultivated at 17°C are identical to those in Fig 1A. C and D. Wild type and *pde-5* animals and *pde-5* mutant animals expressing PDE-5 in AFD that express GCaMP3 Ca^2+^ indicator and tagRFP in AFD were cultivated at 17°C or 23°C. Animals were then subjected to imaging analysis with temperature stimuli indicated (orange line). Individual (gray) and average (pea green) fluorescence ratio (GCaMP/RFP) change at AFD sensory ending (C) and soma (D) is shown. E. *pde-5(nj49); njEx1414[gcy-8p*::*pde-5*::*GFP*, *ges-1p*::*NLStagRFP]* was subjected to microscopic analysis with Zeiss LSM880 confocal microscope.

*pde-5* is expressed in AFD [29]. To examine whether *pde-5* acts in AFD to regulate cGMP dynamics and thermotaxis, we expressed *pde-5* specifically in AFD in *pde-5* mutants. AFD-specific *pde-5* expression significantly rescued abnormality in thermotaxis of *pde-5* mutants cultivated at 23°C at least (Fig 4A), indicating that *pde-5* acts in AFD to regulate thermotaxis. We therefore monitored AFD cGMP dynamics in *pde-5* mutants. Although cGMP level is supposed to increase in *pde-5* mutants that lack a phosphodiesterase, cGMP dynamics at AFD sensory ending seemed abolished in *pde-5* mutant animals cultivated at both 17°C and 23°C (Fig 4B). Abolished cGMP dynamics in *pde-5* mutants were rescued by AFD-specific expression of *pde-5* (Fig 4B), suggesting that *pde-5* cell-autonomously acts in AFD to regulate cGMP dynamics and thereby thermotaxis behavior. Expression of PDE-5 fused to GFP was observed throughout AFD including its sensory ending, indicating that PDE-5 may well affect cGMP dynamics at AFD sensory ending (Fig 4E).

We next aimed to examine how the abolished cGMP dynamics affected on Ca^2+^ dynamics in *pde-5* mutants. Surprisingly, the Ca^2+^ dynamics in *pde-5* mutants were indistinguishable from that in wild type (Fig 4C and 4D). Given that the Ca^2+^ dynamics are supposed to be downstream of the cGMP dynamics, it seems paradoxical that the cGMP dynamics were abolished, while the Ca^2+^ dynamics were intact, and nevertheless thermotaxis was abnormal in *pde-5* mutants. One possible explanation for the discrepancy between the abolished cGMP dynamics and the intact Ca^2+^ dynamics is that cGMP concentration in *pde-5* mutants were out of cGi-500’s operating range due to high basal cGMP level caused by inadequate cGMP hydrolysis or to small amplitude, and the Ca^2+^ dynamics reflected that undetected cGMP dynamics. To examine if the basal cGMP level is increased in *pde-5*, R_0_ was analyzed (S4C Fig). However, R_0_ in *pde-5* did not significantly exceed that of wild type. We are not completely sure yet whether R_0_ properly reflect the basal cGMP level since R_0_ was not decreased in *gcy-23 gcy-8 gcy-18* triple mutants, where cGMP is supposed to be decreased. The discrepancy between the normal Ca^2+^ dynamics and the abnormal thermotaxis could be possibly because the abnormality in Ca^2+^ dynamics was not prominent enough with the used temperature stimuli.

### *pde-1* and *pde-2* act in AFD to regulate thermotaxis

*pde-1* and *pde-2* are also expressed in AFD [29, 34]. AFD-specific expression of *pde-1* or *pde-2* rescued abnormality in thermotaxis of *pde-1; pde-2* double mutants at least in part to the comparable level of *pde-2* or *pde-1* single mutants (S5A Fig). These results indicate that both *pde-1* and *pde-2* act in AFD to regulate thermotaxis. We therefore monitored cGMP and Ca^2+^ dynamics in AFD of *pde-1*, *pde-2* and *pde-1; pde-2* mutant animals. Unexpectedly, both cGMP and Ca^2+^ dynamics in these mutants were indistinguishable from those in wild type (S5B–S5D Fig). The difference of cGMP and Ca^2+^ dynamics that caused the abnormal thermotaxis could be possibly not prominent enough with the used temperature stimuli. It is also possible that *pde-1* and *pde-2* might function downstream of Ca^2+^ increment to regulate thermotaxis, for instance, by regulating transmission from AFD to a downstream interneuron [35–37].

### *pde-1* and *pde-5* synergize in AFD

**Fig 5:**
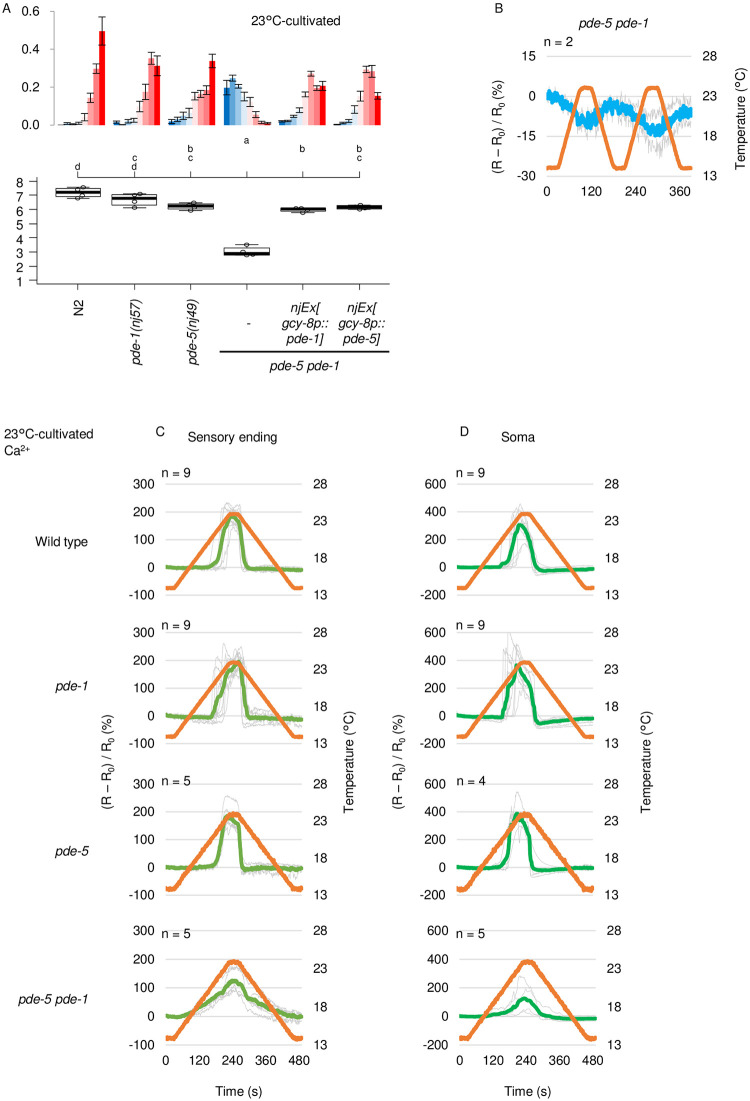
*pde-1* and *pde-5* synergize in AFD. A. Wild type, *pde-1*, *pde-5* and *pde-5 pde-1* double mutant animals and *pde-5 pde-1* animals that express *pde-1* or *pde-5* in AFD were cultivated at 23°C and then subjected to thermotaxis assay. n = 4. The error bars in histograms represent the standard error of mean (SEM). The thermotaxis indices of strains marked with distinct alphabets differ significantly (p < 0.05) according to the Tukey-Kramer test. B. *pde-5 pde-1* mutant animals that express cGi-500 cGMP indicator in AFD were cultivated at 23°C and subjected to imaging analysis. C-D. Wild type, *pde-1*, *pde-5* and *pde-5 pde-1* double mutant animals that express GCaMP3 Ca^2+^ indicator and tagRFP in AFD were cultivated at 23°C and subjected to imaging analysis. Warming and cooling was at the rate of 1°C/20 sec. Individual (gray) and average (pea green or green) fluorescence ratio (GCaMP/RFP) change at AFD sensory ending (C) and soma (D) is shown. Data of wild type and *pde-1* animals are identical to those in S5C and S5D Fig.

Mutations in both *pde-5* and *pde-1* genes exhibited a synergistic effect on thermotaxis especially when cultivated at 23°C (Fig 3). We then examined whether the synergistic effect of *pde-1* and *pde-5* mutations is derived from the cell-autonomous acts of these genes in AFD. AFD-specific expression of *pde-1* or *pde-5* partially rescued abnormality of *pde-5 pde-1* double mutants to the level of *pde-1* or *pde-5* single mutants (Fig 5A). Not surprisingly, cGMP dynamics in *pde-5 pde-1* double mutants seemed abolished similarly in *pde-5* mutants (Figs 4B and 5B). Significant elevation of the baseline (R0) was also not detected in *pde-5 pde-1* (S4C Fig). Interestingly, whereas Ca^2+^ dynamics in *pde-1* or *pde-5* single mutants were almost comparable to that in wild type, in *pde-5 pde-1* double mutants, Ca^2+^ gradually increased from lower temperature than the wild type and decreased gradually (Fig 5C and 5D). These results suggest that *pde-1* and *pde-5* redundantly function in AFD, possibly by hydrolyzing cGMP that temporally or spatially should not exist, to suppress Ca^2+^ increment in response to warming below threshold, to sharply decrease Ca^2+^ in response to cooling, and therefore to limit the temperature range to which AFD responds, which is essential for proper thermotaxis.

## Discussion

**Fig 6:**
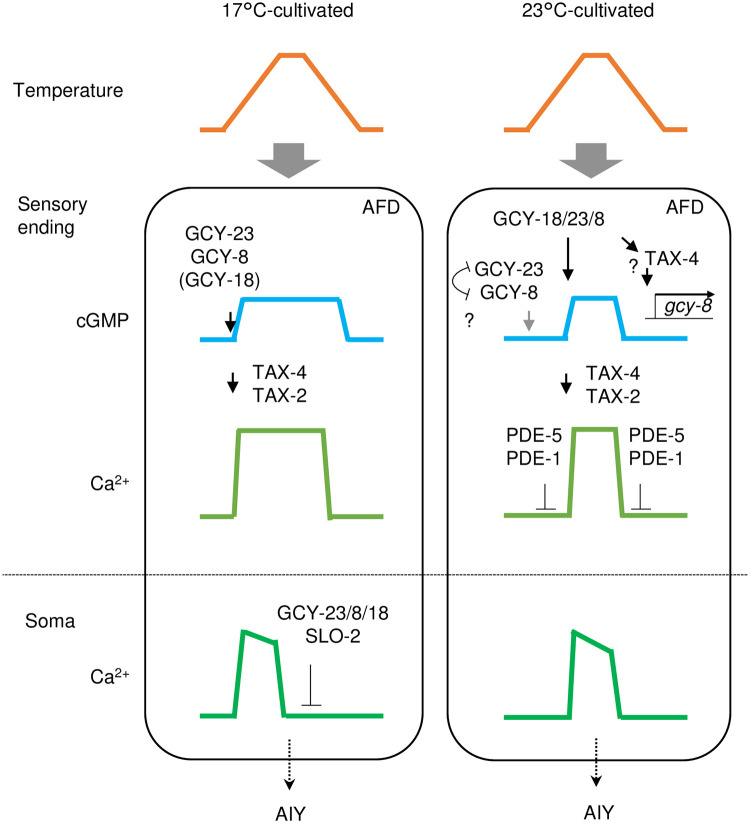
Contribution of GCYs and PDEs on cGMP and Ca^2+^ dynamics in AFD cultivated at different temperature. In animals cultivated at 17°C, any of GCY-23, GCY-8 and GCY-18 can probably contribute to the cGMP increment in response to warming since all of three *gcy* double mutants show Ca^2+^ response (S2 and S3 Figs). cGMP production by GCY-18 alone might not be sufficient since *gcy-23 gcy-8* shows slightly defective thermotaxis (Fig 2A). Ca^2+^ level in soma is actively decreased via the three GCYs (S3 Fig) and SLO-2 potassium channel [40]. In animals cultivated at 23°C, activity of GCY-23 and GCY-8 is suppressed below the threshold temperature by coexistence of both, possibly forming an inactive dimer. Threshold for GCY-18 seems to be adjustable by an unknow AFD-specific mechanism (See ’Discussion‘ section). Transcription of *gcy-18* is increased under higher cultivation temperature [40, 41]. Transcription of *gcy-8* might be regulated by GCY-18 and TAX-4 (S4A Fig). Importantly, PDE-5 and PDE-1 collaborate to suppress Ca^2+^ level under threshold temperature, which seems to be essential for thermotaxis.

In this study, we demonstrated that cGMP increases and decreases in AFD thermosensory neurons of *C*. *elegans* in response to warming and cooling, respectively (Fig 1). These cGMP dynamics were observed specifically at sensory endings but not at soma, which contrasted with Ca^2+^ dynamics that are uniform among subcompartments in AFD such as sensory ending and soma [12] (S1 Fig). Given that GCYs and TAX-2 and TAX-4 CNG channels localize at the sensory ending in AFD and that Ca^2+^ dynamics are uniform all over AFD, a mechanism that propagates Ca^2+^ increment from the sensory endings is supposed to exist; such as EGL-19 voltage-gated Ca^2+^ channel functioning in the ASEL chemosensory neuron [38]. We also observed that the cGMP dynamics, which are supposed to precede Ca^2+^ dynamics, already reflected the comparison between memorized past T_c_ and present ambient temperature consistently with a previous report [24]. We further showed that AFD-specific GCYs determine onset temperature for cGMP increment, which is reflected on Ca^2+^ dynamics and thermotaxis behavior (Fig 2, S1 and S2 Figs). Moreover, we described complex roles of PDEs in forming cGMP and Ca^2+^ dynamics and in thermotaxis behavior (Figs 3–6 and S5 Fig).

Intriguingly, we observed in several experiments that AFD Ca^2+^ level already start decreasing at the temperature range where cGMP level was constant, which was more prominent in soma rather than in sensory endings (Fig 4B–4D, wild type, 17°C-cultivated) and particularly evident in *gcy-8 gcy-18* and *gcy-23 gcy-18* double mutant animals cultivated at 23°C (Fig 2B and S2A, S2B, S3A and S3B Figs right). In contrast, cGMP level began to decrease almost at the same temperature as the onset temperature for increment (Fig 2B–2D). Given the off time constant of cGi-500 after cGMP decrement (less than 80 ms) is smaller than that of GCaMP3 after Ca^2+^ decrement (less than 1 sec) [25, 39], this is not likely to be due to the slow decay of cGi-500 fluorescence. Therefore, it seems that mechanisms that prevents Ca^2+^ influx and/or decrease Ca^2+^ level despite high cGMP level exist in AFD, which is consistent with our previous finding that SLO K^+^ channels contribute to decrement of Ca^2+^ level in AFD after its increment by warming [40]; both SLO-1 and SLO-2 channels are activated by Ca^2+^, and the resulting K+ efflux may prevent Ca^2+^ influx possibly by inactivating voltage-dependent Ca^2+^ channels. Interestingly, all the three *gcy* double mutants cultivated at 17°C showed prolonged Ca^2+^ signal at soma (S2 Fig, left), suggesting that the full set of GCYs is necessary for the Ca^2+^ shutdown (Fig 6), although this defective Ca^2+^ dynamics did not affect thermotaxis so much (Fig 2A, upper).

cGMP and Ca^2+^ increase stereotypically in AFD in response to warming according to past T_c_, however, it remains elusive how the past T_c_ is memorized as the onset temperature for cGMP and Ca^2+^ increment. Interestingly, when GCY-18 are ectopically expressed in chemosensory neurons, they confer fixed onset temperature for Ca^2+^ increment regardless of animals’ T_c_ [20]. In contrast, onset temperature for Ca^2+^ increment in AFD of *gcy-23 gcy-8* double mutants, in which only GCY-18 is expressed out of the three GCYs essential for the AFD thermosensation, was changeable according to T_c_ (S2 & S3 Figs). Consistently, thermotaxis of *gcy-23 gcy-8* double mutants was changeable according to T_c_ (Fig 2). The adjustability of AFD with only GCY-18 regarding its onset temperature, which was not achieved by ectopic expression of GCY-18 in chemosensory neurons, could be possibly explained in two ways: First, a mechanism that assists GCY-18 to be activated at lower temperature exists in AFD, or though less likely a mechanism that inhibits GCY-18 to be activated at lower temperature exists in chemosensory neurons. Second, given that expression level of *gcy* mRNAs change according to T_c_ [40, 41], regulation of GCY-18 expression level through *gcy-18* promoter, which was not involved during the ectopic GCY-18 expression in chemosensory neurons [20], might be necessary for the adjustability of AFD onset temperature in *gcy-23 gcy-8* double mutants. Apart from the transcriptional regulation leading to the change in GCY expression ratio, it is possible and yet to be determined that the three GCYs with distinct characteristics make heterodimers that have distinct characteristics from each homodimer. If that is the case, these variety of elements might fine-tune the responsiveness of AFD according to T_c_. Given GCY-23 and GCY-8 are active below T_c_ when existing alone but their activity is probably suppressed when they co-exist (Fig 2 and S4B Fig), GCY-23 and GCY-8 might form an inactive heterodimer below T_c_ (Fig 6).

The sensory ending of AFD has a unique and complicated morphology with microvilli [30, 34, 42], and the GCYs’ localization is restricted in the sensory ending including these microvilli by genes related to ciliary function [16, 31]. Loss of cilia during evolution is well correlated with loss of cGMP signaling pathway, suggesting that ciliary function and cGMP signaling are interdependent [43]. Given that *C*. *elegans* mutants for the cilia-related genes are defective for thermotaxis [31, 44], GCYs’ localization in the sensory ending may contribute to AFD’s ability to encode T_c_ with cGMP and Ca^2+^ dynamics and therefore to thermotaxis.

We showed that PDE-1, PDE-2 and PDE-5 act in AFD to regulate thermotaxis. However, contributions of those PDEs to cGMP and Ca^2+^ dynamics and to thermotaxis did not seem to be so straight-forward as those of GCYs. How these PDEs, especially PDE-5, affect cGMP dynamics needs to be re-evaluated once a cGMP indicator with improved sensitivity and/or dynamic range is developed. Given that the loss of *pde-1* together with *pde-5* showed synergistic effect both on Ca2+ dynamics and on behavior (Fig 5), cGMP dynamics in *pde-5 pde-1* double mutants might be different from that in *pde-5*, although it seemed totally abolished in both strains during our measurements possibly due to the limited sensitivity of the probe. Nevertheless, we here found that PDE-1 and PDE-5 redundantly function to restrict the dynamic range of AFD’s Ca^2+^ response, which is supposed to be essential for proper thermotaxis behavior (Fig 6).

## Materials and methods

### Experimental model and subject details

*C*. *elegans* strains were cultivated on nematode growth medium (NGM) plates seeded with *E*. *Coli* OP50 strain (Caenorhabditis Genetics Center (CGC), Twin Cities, MN, USA) as described [45]. N2 (Bristol) was used as the wild type strain unless otherwise indicated. Transgenic lines were generated by injecting plasmid DNA directly into hermaphrodite gonad as described [46]. Strains used in this study were listed in S1 Table.

### Behavioral assays

Population thermotaxis (TTX) assays were performed as described previously [47]. Briefly, 50 to 250 animals cultivated at 17°C or 23°C were placed on the center of the assay plates without food with the temperature gradient of 17–23°C and were allowed to freely move for 1 h. The assay plate was divided into eight sections along the temperature gradient, and the number of the adult animals in each section was scored. Ratio of animal numbers in each section was plotted in histograms. Thermotaxis indices were calculated as shown below:

$$\frac{\sum_{i=1}^{8}i∙N_{i}}{N}$$

N_i_: number of animals in each section i (i = 1 to 8), N: total number of animals on the test plate.

### Plasmids

pAF-EXPR-26 *gcy-37p*::*cGi-500*::*unc-54 3’UTR* was a gift from Dr. de Bono [33]. cGi-500 was sub-cloned into pDONR221 using the Gateway BP reaction (Thermo Fisher Scientific, Waltham, MA, USA). To create a plasmid to express cGi-500 in AFD, gcy-8 promoter, cGi-500 cDNA and *unc-54* 3’UTR were fused by MultiSite Gateway Technology (Thermo Fisher Scientific, Waltham, MA, USA).

*pde-1b*, *pde-2a* and *pde-5* cDNAs were PCR amplified from DupLEX-A Yeast Two-Hybrid cDNA library *C*. *elegans* (adult) (OriGene, Rockville, MD) and cloned into AgeI-NotI site of pIA138 *gcy-8p*::*VN173*::*unc-54 3’UTR*.

Plasmids used in this study are listed in S2 Table. Details regarding the plasmid constructs including sequences can be obtained from the authors.

### Imaging analyses

cGMP and Calcium imaging was performed as described elsewhere [15, 40]. Briefly, a single adult animal that expressed genetically encoded cGMP indicator cGi-500 [25] or that express calcium indicator GCaMP3 [48] and tagRFP in AFD was placed on a 10% agar pad on a cover slip with 0.1 μm polystyrene beads (Polysciences, Warrington, PA, USA) and covered by another cover slip for immobilization [49]. The immobilized animals were placed on a Peltier-based temperature controller (Tokai Hit, Fujinomiya, Japan) on a stage of BX61WI microscope (Olympus, Tokyo, Japan). The cyan and yellow, or red and green fluorescence was separated by the Dual-View optics system (Teledyne photometrics, AZ, USA), and the images were captured by an EM-CCD camera C9100-13 ImageEM (Hamamatsu Photonics, Hamamatsu, Japan) at 1 Hz frame rate. Excitation pulses were generated by SPECTRA light engine (Lumencor, Beaverton, OR, USA). The fluorescence intensities were measured by the MetaMorph imaging system (Molecular Devices). Change of fluorescence ratio R (CFP/YFP for cGMP imaging with cGi-500 and GFP/RFP for Ca^2+^ imaging with GCaMP3 and tagRFP), (R–R_0_) / R_0_, was plotted, where R_0_ is average of R from t = 1 to t = 31.

Expression of PDE-5::GFP was observed with LSM880 confocal microscope (Zeiss, Oberkochen, Germany).

### Statistical analysis

The error bars in histograms for thermotaxis assays indicate the standard error of mean (SEM). In the boxplots, the bottom and top of boxes represent the first and third quartiles, and the band inside the box represents the median. The ends of the lower and upper whiskers represent the lowest datum still within the 1.5 interquartile range (IQR), which is equal to the difference between the third and first quartiles, of the lower quartile, and the highest datum still within the 1.5 IQR of the upper quartile, respectively. For multiple-comparison tests, one-way analyses of variance (ANOVAs) were performed, followed by Tukey-Kramer test or Dunnett test. Statistic analyses were done by R programming language.

## Supporting information

S1 Fig Extraction of onset temperature for cGMP increment and decrement (related to Fig 2). Individual traces of fluorescence ratio (CFP/YFP) change shown in Fig 2B were subjected to analysis to extract time points at which the mean of fluorescence ratio change changes most significantly by using MATLAB command ‘findchangepts’. Extracted time points were indicated below the traces, among which those regarded as beginning points for increment in response to the warming and decrement in response to the 2^nd^ cooling were made bold and bold-italic, respectively.
(TIF)

S2 Fig Ca^2+^ onsets from lower temperature in AFD sensory ending of gcy double mutants (related to Fig 2). Wild type and *gcy* double mutant animals indicated that express GCaMP3 Ca^2+^ indicator and tagRFP in AFD were cultivated at 17°C (left) or 23°C (right) and subjected to imaging analysis with temperature stimuli indicated (orange line). Warming and cooling was at the rate of 1°C/20 sec. n = 4 to 6. Individual (gray) and average fluorescence ratio (GCaMP/RFP) change at AFD sensory ending is shown. B. Temperature at which Ca^2+^ level started increasing in response to warming was extracted using a MATLAB command ‘findchangepts’ and plotted. p values were indicated, or *** indicates p < 0.001 (Dunnett test against wild type animals).
(TIF)

S3 Fig Ca^2+^ onsets from lower temperature in AFD soma of *gcy* double mutants (related to Fig 2). Wild type and *gcy* double mutant animals indicated that express GCaMP3 Ca^2+^ indicator and tagRFP in AFD were cultivated at 17°C (left) or 23°C (right) and subjected to imaging analysis with temperature stimuli indicated (orange line). Warming and cooling was at the rate of 1°C/20 sec. n = 4 to 6. Individual (gray) and average fluorescence ratio (GCaMP/RFP) change at AFD soma is shown. B. Temperature at which Ca^2+^ level started increasing in response to warming was extracted using a MATLAB command ‘findchangepts’ and plotted. p values were indicated, or *** indicates p < 0.001 (Dunnett test against wild type animals).
(TIF)

S4 Fig Basal YFP fluorescence and CFP/YFP fluorescence ratio (R_0_) of cGi-500 (related to Figs 1, 2, 4 and 5, S5 Fig). A. Mean values of YFP fluorescence intensity between t = 0 and t = 31, while temperature was kept constantly at 23°C, were plotted for 23°C-cultivated animals of indicated genotype expressing cGi-500 cGMP indicator specifically in AFD thermosensory neurons used in Fig 2B. *** indicates p < 0.001 (Dunnett test against wild type animals). B. Mean values of CFP/YFP fluorescence ratio between t = 0 and t = 31 for the same measurement in A were plotted. p values were indicated (Dunnett test against wild type animals). C. Mean values of CFP/YFP fluorescence ratio between t = 0 and t = 31, while temperature was kept constantly at 14°C, were plotted for 23°C-cultivated animals of indicated genotype used in Figs 1, 4B and 5B, S5B Fig. p values were indicated (Tukey-Kramer test).
(TIF)

S5 Fig *pde-1* and *pde-2* act in AFD to regulate thermotaxis. A. Wild type, *pde-1*, *pde-2* and *pde-1; pde-2* animals and *pde-1; pde-2* animals that express PDE-1 or PDE-2 specifically in AFD were cultivated at 17°C or 23°C and then subjected to thermotaxis assay. n = 8 for N2 and *pde-1; pde-2*. n = 4 for others. The error bars in histograms represent the standard error of mean (SEM). The thermotaxis indices of strains marked with distinct alphabets differ significantly (p < 0.05) according to the Tukey-Kramer test. B. Wild type and mutant animals lacking *pde* gene(s) indicated that express cGi-500 cGMP indicator in AFD were cultivated at 23°C and subjected to imaging analysis. Warming and cooling was at the rate of 1°C/6 sec. Individual (gray) and average (blue) fluorescence ratio (CFP/YFP) change at AFD sensory ending is shown. C-D. Wild type and mutant animals lacking *pde* gene(s) indicated that express GCaMP3 Ca^2+^ indicator and tagRFP in AFD were cultivated at 23°C and subjected to imaging analysis. Warming and cooling was at the rate of 1°C/20 sec. Individual (gray) and average (pea green or green) fluorescence ratio (GCaMP/RFP) change at AFD sensory ending (C) and soma (D) is shown.
(TIF)

S1 Table Strain list. (DOCX)

S2 Table Plasmid list. (DOCX)
