# Correction: cGMP dynamics that underlies thermosensation in temperature-sensing neuron regulates thermotaxis behavior in C. elegans

**DOI:** 10.1371/journal.pone.0305045

**Published:** 2024-05-31

**Authors:** The PLOS ONE Staff

## Notice of Republication

This article was republished on May 20, 2024, to correct an error in the author list: Ichiro Aoki was erroneously displayed as Ichiro Aok. The publisher apologizes for the error. Please download this article again to view the correct version. The originally published, uncorrected article and the republished, corrected article are provided here for reference.

## Supporting information

S1 File Originally published, uncorrected article. (PDF)

S2 File Republished, corrected article. (PDF)
