# Supplementary figures and images for: cGMP dynamics that underlies thermosensation in temperature-sensing neuron regulates thermotaxis behavior in C. elegans
Source: PLoS One. 2022 Dec 6;17(12):e0278343. doi: 10.1371/journal.pone.0278343 (PMC9725164; doi:10.1371/journal.pone.0278343)

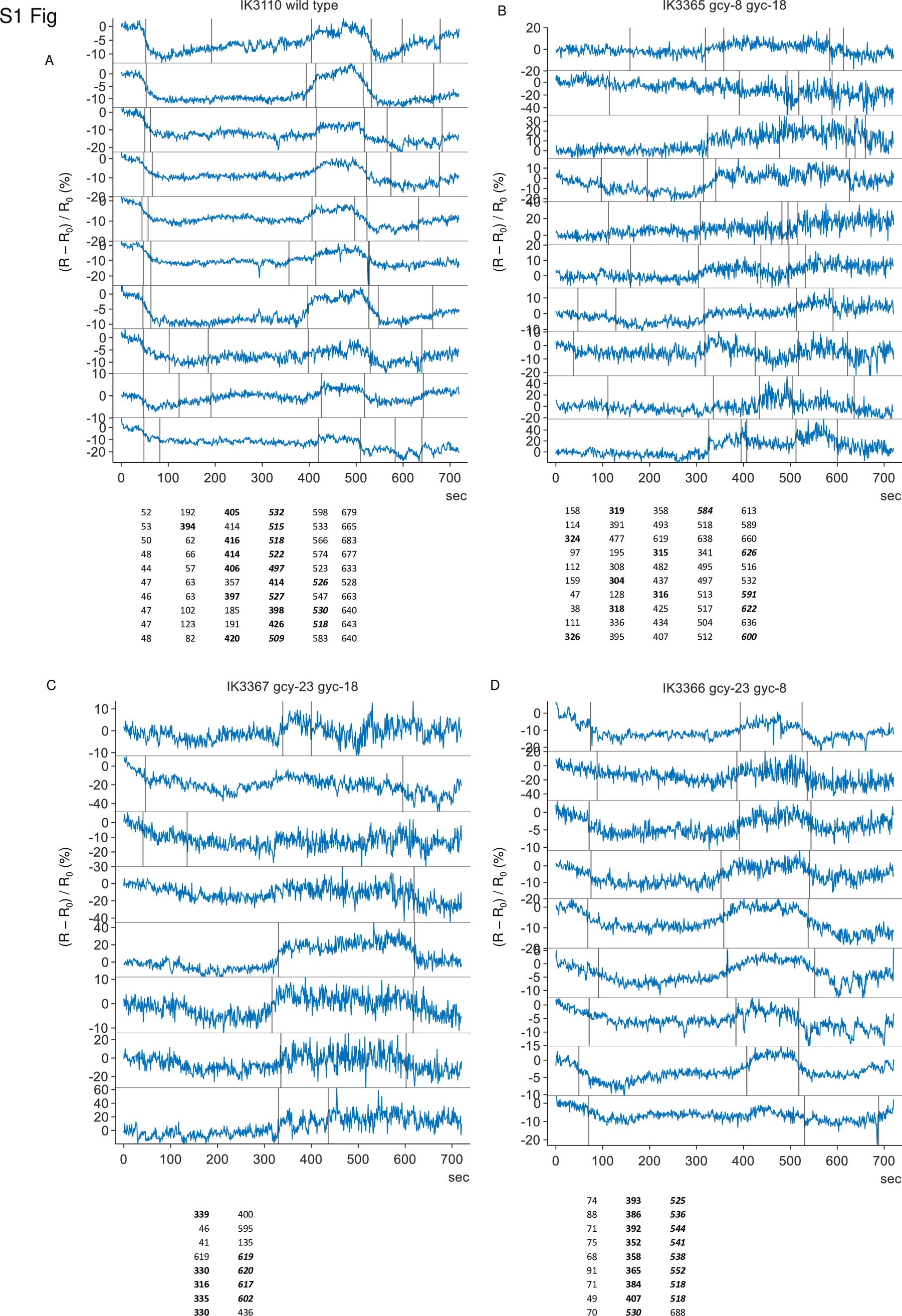

Supplement: S1 Fig — Individual traces of fluorescence ratio (CFP/YFP) change shown in Fig 2B were subjected to analysis to extract time points at which the mean of fluorescence ratio change changes most significantly by using MATLAB command ‘findchangepts’. Extracted time points were indicated below the traces, among which those regarded as beginning points for increment in response to the warming and decrement in response to the 2nd cooling were made bold and bold-italic, respectively. (TIF) [file pone.0278343.s001.tif]

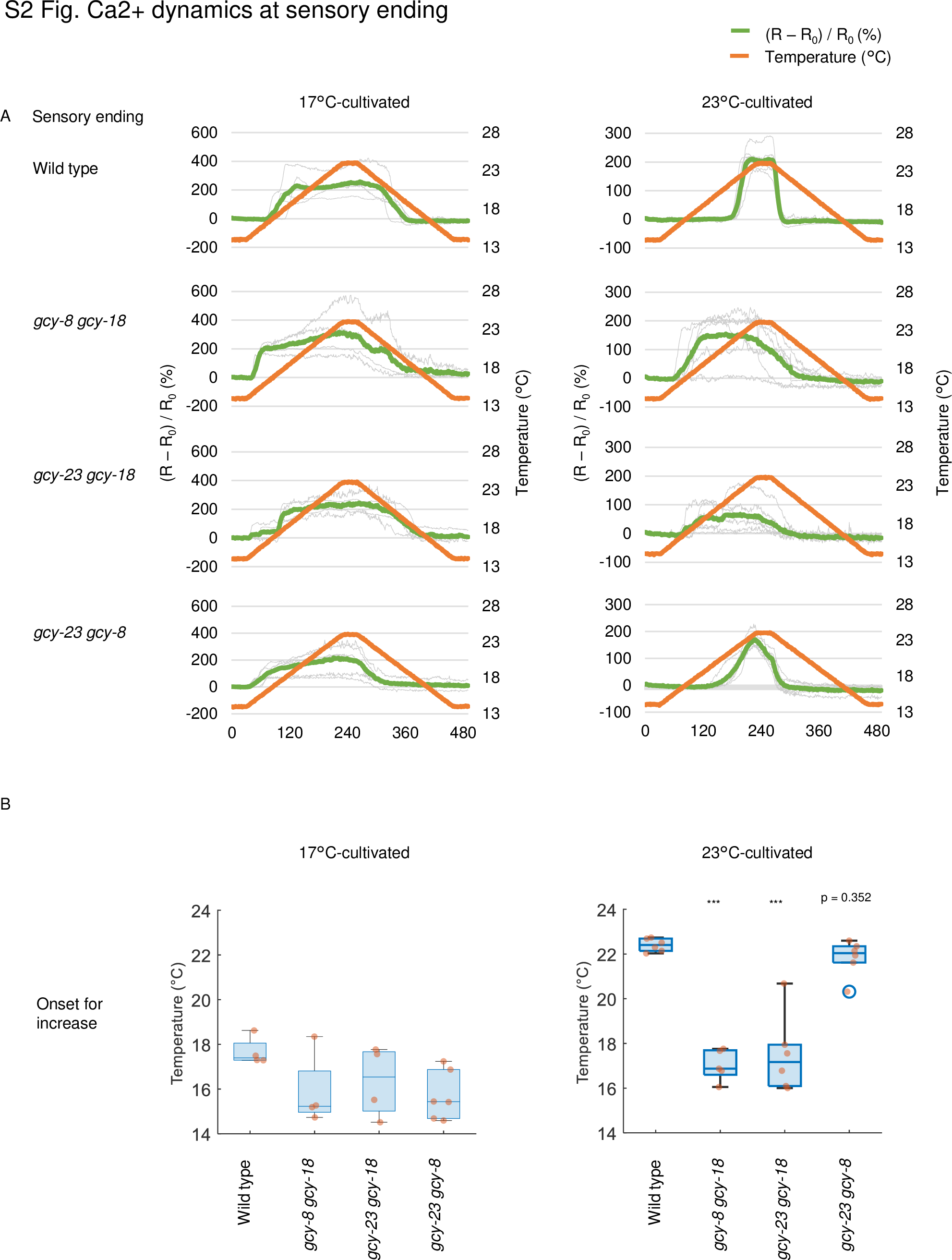

Supplement: S2 Fig — Wild type and gcy double mutant animals indicated that express GCaMP3 Ca2+ indicator and tagRFP in AFD were cultivated at 17°C (left) or 23°C (right) and subjected to imaging analysis with temperature stimuli indicated (orange line). Warming and cooling was at the rate of 1°C/20 sec. n = 4 to 6. Individual (gray) and average fluorescence ratio (GCaMP/RFP) change at AFD sensory ending is shown. B. Temperature at which Ca2+ level started increasing in response to warming was extracted using a MATLAB command ‘findchangepts’ and plotted. p values were indicated, or *** indicates p < 0.001 (Dunnett test against wild type animals). (TIF) [file pone.0278343.s002.tif]

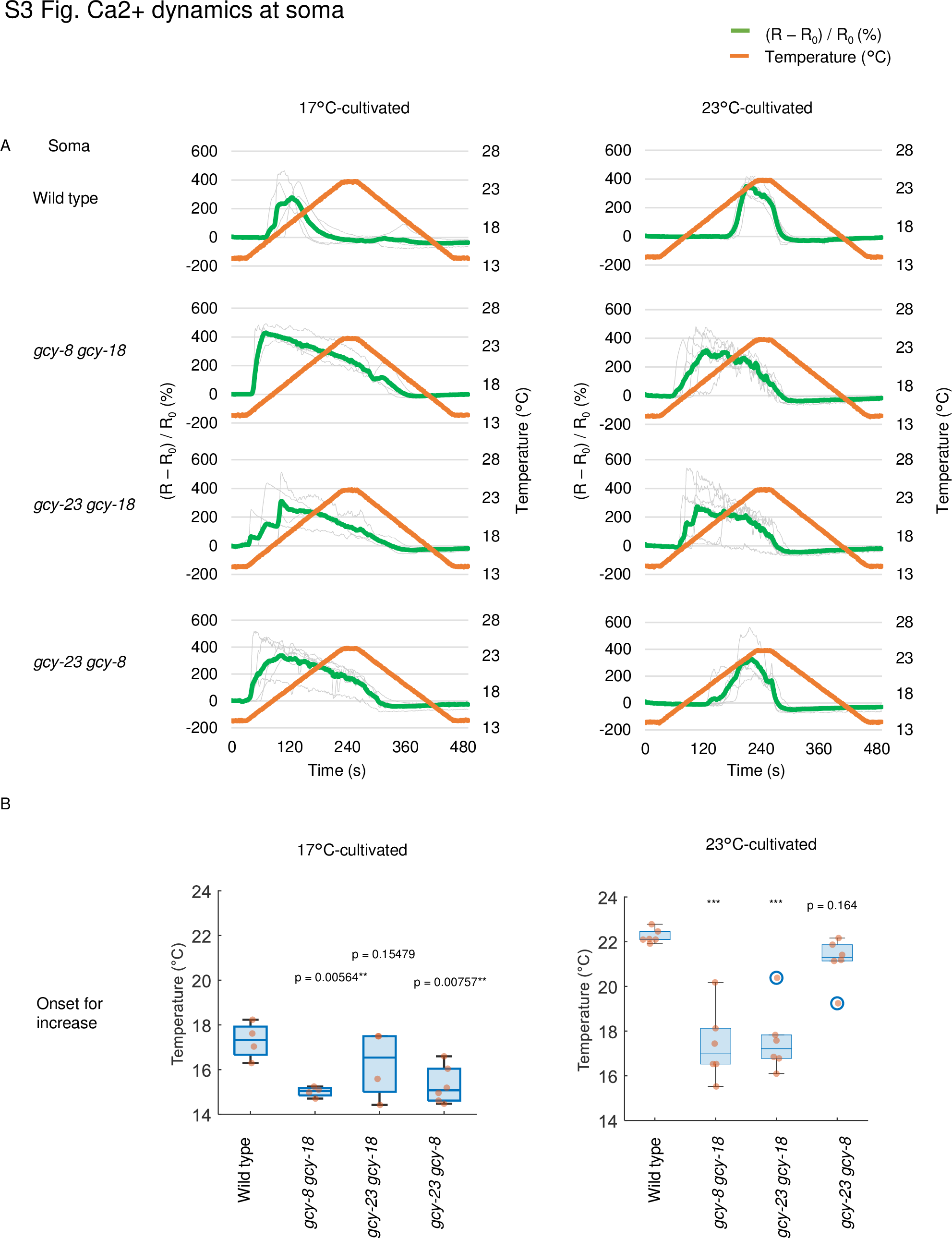

Supplement: S3 Fig — Wild type and gcy double mutant animals indicated that express GCaMP3 Ca2+ indicator and tagRFP in AFD were cultivated at 17°C (left) or 23°C (right) and subjected to imaging analysis with temperature stimuli indicated (orange line). Warming and cooling was at the rate of 1°C/20 sec. n = 4 to 6. Individual (gray) and average fluorescence ratio (GCaMP/RFP) change at AFD soma is shown. B. Temperature at which Ca2+ level started increasing in response to warming was extracted using a MATLAB command ‘findchangepts’ and plotted. p values were indicated, or *** indicates p < 0.001 (Dunnett test against wild type animals). (TIF) [file pone.0278343.s003.tif]

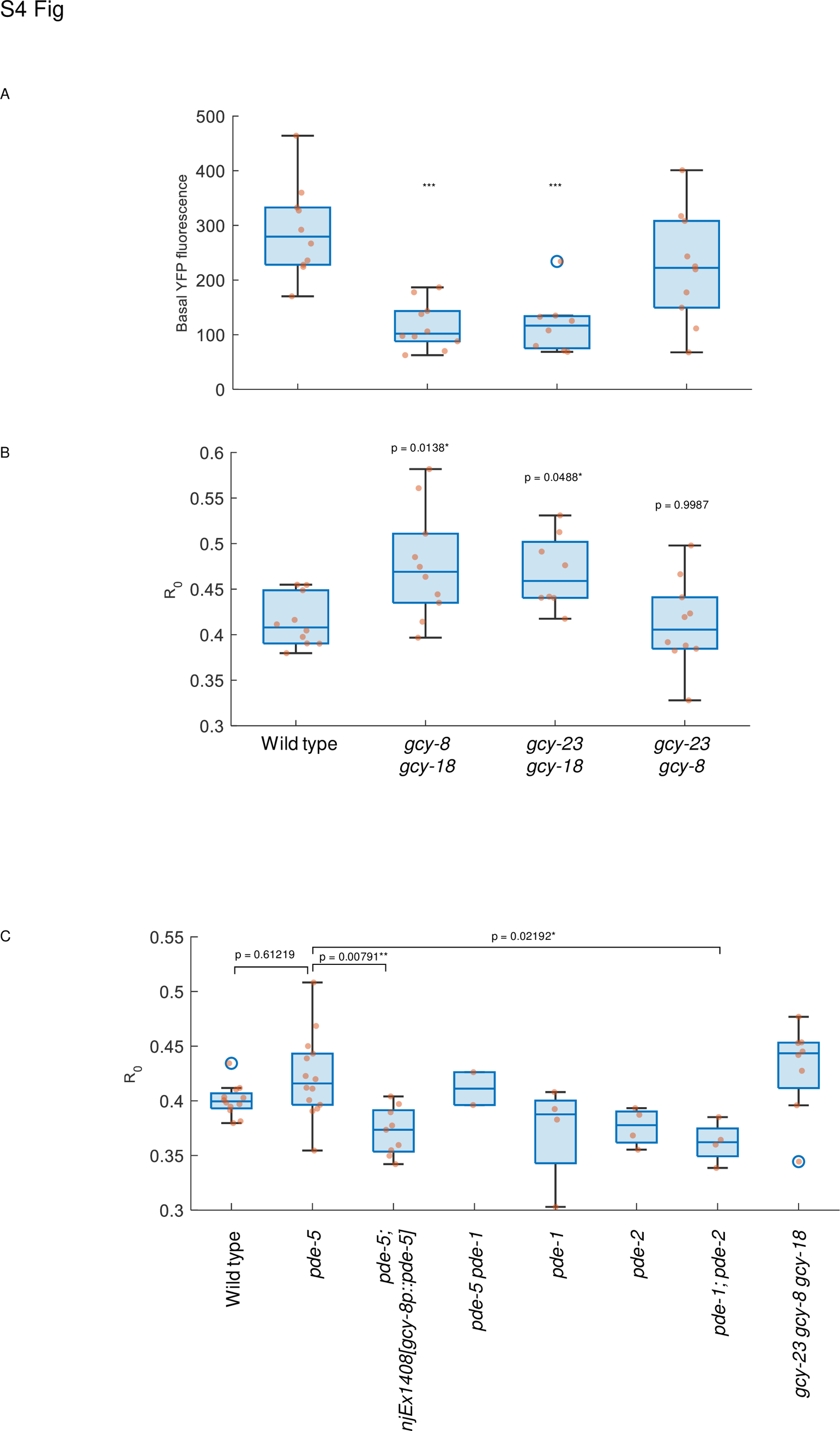

Supplement: S4 Fig — A. Mean values of YFP fluorescence intensity between t = 0 and t = 31, while temperature was kept constantly at 23°C, were plotted for 23°C-cultivated animals of indicated genotype expressing cGi-500 cGMP indicator specifically in AFD thermosensory neurons used in Fig 2B. *** indicates p < 0.001 (Dunnett test against wild type animals). B. Mean values of CFP/YFP fluorescence ratio between t = 0 and t = 31 for the same measurement in A were plotted. p values were indicated (Dunnett test against wild type animals). C. Mean values of CFP/YFP fluorescence ratio between t = 0 and t = 31, while temperature was kept constantly at 14°C, were plotted for 23°C-cultivated animals of indicated genotype used in Figs 1, 4B and 5B, S5B Fig. p values were indicated (Tukey-Kramer test). (TIF) [file pone.0278343.s004.tif]

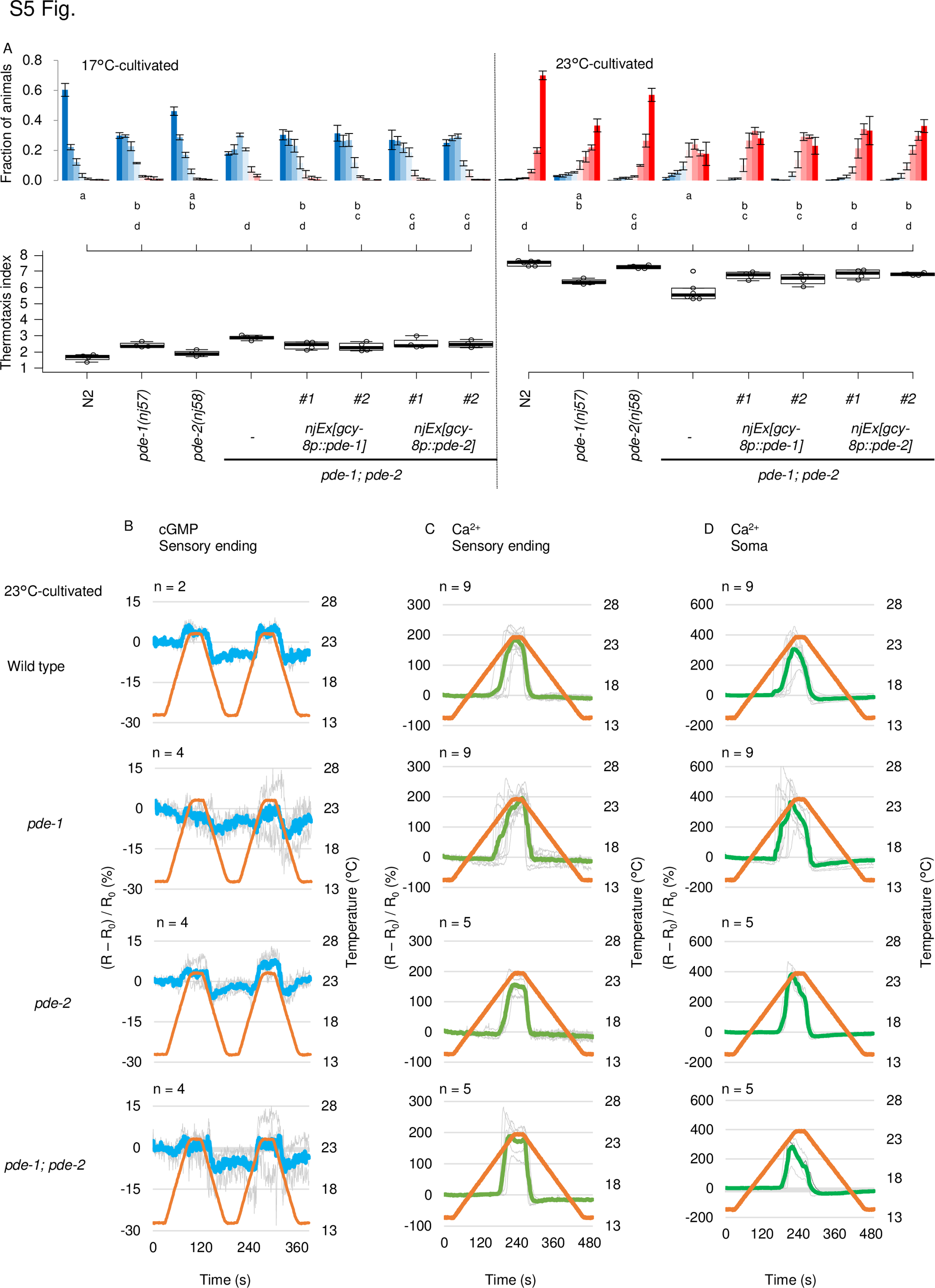

Supplement: S5 Fig — A. Wild type, pde-1, pde-2 and pde-1; pde-2 animals and pde-1; pde-2 animals that express PDE-1 or PDE-2 specifically in AFD were cultivated at 17°C or 23°C and then subjected to thermotaxis assay. n = 8 for N2 and pde-1; pde-2. n = 4 for others. The error bars in histograms represent the standard error of mean (SEM). The thermotaxis indices of strains marked with distinct alphabets differ significantly (p < 0.05) according to the Tukey-Kramer test. B. Wild type and mutant animals lacking pde gene(s) indicated that express cGi-500 cGMP indicator in AFD were cultivated at 23°C and subjected to imaging analysis. Warming and cooling was at the rate of 1°C/6 sec. Individual (gray) and average (blue) fluorescence ratio (CFP/YFP) change at AFD sensory ending is shown. C-D. Wild type and mutant animals lacking pde gene(s) indicated that express GCaMP3 Ca2+ indicator and tagRFP in AFD were cultivated at 23°C and subjected to imaging analysis. Warming and cooling was at the rate of 1°C/20 sec. Individual (gray) and average (pea green or green) fluorescence ratio (GCaMP/RFP) change at AFD sensory ending (C) and soma (D) is shown. (TIF) [file pone.0278343.s005.tif]
