# Supplementary material for: cGMP dynamics that underlies thermosensation in temperature-sensing neuron regulates thermotaxis behavior in C. elegans
Source: PLoS One. 2022 Dec 6;17(12):e0278343. doi: 10.1371/journal.pone.0278343 (PMC9725164; doi:10.1371/journal.pone.0278343)
Supplement: S1 Table — (DOCX) [file pone.0278343.s006.docx]

**S1 Table.** Strain list.

| **Description** | **Source** | **Identifier** |
| --- | --- | --- |
| *C. elegans: wild isolate* | CGC | WormBase: N2  RRID:WB-STRAIN:N2_(ancestral) |
| *pde-1(nj57)* |  | IK0616 |
| *pde-2(nj58)* |  | IK0618 |
| *pde-3(nj59)* |  | IK0620 |
| *pde-5(nj49)* |  | IK0510 |
| *pde-1(nj57); pde-2(nj58)* |  | IK0792 |
| *pde-5(nj49) pde-1(nj57)* |  | IK0794 |
| *pde-5(nj49); pde-2(nj58)* |  | IK0796 |
| *pde-5(nj49) pde-1(nj57); pde-2(nj58)* |  | IK0798 |
| *pde-3(nj59); pde-2(nj58)* |  | IK1395 |
| *pde-5(nj49) pde-1(nj57); pde-3(nj59)* |  | IK1400 |
| *pde-5(nj49) pde-1(nj57); pde-3(nj59); pde-2(nj58)* |  | IK1401 |
| *pde-5(nj49); njEx1408[gcy-8p::pde-5 cDNA, ges-1p::GFP] line1* |  | IK3352 |
| *pde-5(nj49); njEx1409[gcy-8p::pde-5 cDNA, ges-1p::GFP] line2* |  | IK3353 |
| *njEx1285[gcy-8p::cGi-500(60 ng/ul), ges-1p::NLStagRFP] line1* |  | IK3110 |
| *pde-5(nj49); njEx1285[gcy-8p::cGi-500, ges-1p::NLStagRFP] line1* |  | IK3410 |
| *pde-5(nj49); njEx1285[gcy-8p::cGi-500, ges-1p::NLStagRFP]; njEx1408[gcy-8p::pde-5 cDNA, ges-1p::GFP]* |  | IK3439 |
| *njEx359[gcy-8p::TagRFP 20ng, gcy-8p::GCaMP3 50ng)]* |  | IK0855 |
| *pde-5(nj49); njEx359[gcy-8p::TagRFP 20ng, gcy-8p::GCaMP3 50ng)]* |  | IK3434 |
| *pde-5(nj49); njEx359[gcy-8p::TagRFP 20ng, gcy-8p::GCaMP3 50ng)]; njEx1408[gcy-8p::pde-5 cDNA, ges-1p::GFP]* |  | IK3451 |
| *pde-5(nj49); njEx1414[gcy-8p::pde-5::GFP, ges-1p::NLStagRFP] line1* |  | IK3358 |
| *pde-1(nj57); pde-2(nj58); njEx1410[gcy-8p::pde-1 cDNA, ges-1p::GFP] line1* |  | IK3354 |
| *pde-1(nj57); pde-2(nj58); njEx1411[gcy-8p::pde-1 cDNA, ges-1p::GFP] line2* |  | IK3355 |
| *pde-1(nj57); pde-2(nj58); njEx1412[gcy-8p::pde-2 cDNA, ges-1p::GFP] line1* |  | IK3356 |
| *pde-1(nj57); pde-2(nj58); njEx1413[gcy-8p::pde-2 cDNA, ges-1p::GFP] line2* |  | IK3357 |
| *pde-1(nj57); njEx1285[gcy-8p::cGi-500, ges-1p::NLStagRFP] line1* |  | IK3407 |
| *pde-2(nj58); njEx1285[gcy-8p::cGi-500, ges-1p::NLStagRFP]* |  | IK3409 |
| *pde-1(nj57); pde-2(nj58); njEx1285[gcy-8p::cGi-500, ges-1p::NLStagRFP]* |  | IK3433 |
| *pde-5(nj49) pde-1(nj57); njEx1285[gcy-8p::cGi-500, ges-1p::NLStagRFP]* |  | IK3440 |
| *pde-5(nj49) pde-1(nj57); njEx359[gcy-8p::TagRFP 20ng, gcy-8p::GCaMP3 50ng)]* |  | IK3453 |
| *pde-1(nj57); njEx359[gcy-8p::TagRFP 20ng, gcy-8p::GCaMP3 50ng)]* |  | IK3452 |
| *pde-5(nj49) pde-1(nj57); njEx1410[gcy-8p::pde-1 cDNA, ges-1p::GFP]* |  | IK3457 |
| *pde-5(nj49) pde-1(nj57); njEx1408[gcy-8p::pde-5 cDNA, ges-1p::GFP]* |  | IK3458 |
| *gcy-23(nj37) gcy-8(oy44) gcy-18(nj38); njEx1285[gcy-8p::cGi-500, ges-1p::NLStagRFP]* |  | IK3360 |
| *gcy-8(oy44) gcy-18(nj38); njEx1285[gcy-8p::cGi-500, ges-1p::NLStagRFP]* |  | IK3365 |
| *gcy-23(nj37) gcy-8(oy44); njEx1285[gcy-8p::cGi-500, ges-1p::NLStagRFP]* |  | IK3366 |
| *gcy-23(nj37) gcy-18(nj38); njEx1285[gcy-8p::cGi-500, ges-1p::NLStagRFP]* |  | IK3367 |
| *gcy-8(oy44) gcy-18(nj38); njEx359[gcy-8p::TagRFP 20ng, gcy-8p::GCaMP3 50ng)]* |  | IK3454 |
| *gcy-23(nj37) gcy-8(oy44); njEx359[gcy-8p::TagRFP 20ng, gcy-8p::GCaMP3 50ng)]* |  | IK3455 |
| *gcy-23(nj37) gcy-18(nj38); njEx359[gcy-8p::TagRFP 20ng, gcy-8p::GCaMP3 50ng)]* |  | IK3456 |
| *tax-4(p678); njEx1285[gcy-8p::cGi-500, ges-1p::NLStagRFP]* |  | IK3412 |
