# Supplementary material for: cGMP dynamics that underlies thermosensation in temperature-sensing neuron regulates thermotaxis behavior in C. elegans
Source: PLoS One. 2022 Dec 6;17(12):e0278343. doi: 10.1371/journal.pone.0278343 (PMC9725164; doi:10.1371/journal.pone.0278343)
Supplement: S2 Table — (DOCX) [file pone.0278343.s007.docx]

**S2 Table.** Plasmid list.

| **Description** | **Source** | **Identifier** |
| --- | --- | --- |
| *gcy-8p::cGi500* | This paper | pMS001 |
| *gcy-8p::pde-1b* | This paper | pIA140 |
| *gcy-8p::pde-2a* | This paper | pIA141 |
| *gcy-8p::pde-5* | This paper | pIA143 |
| *gcy-8p::pde-5::gfp* | This paper | pMS005 |
