# Supplementary material for: cGMP dynamics that underlies thermosensation in temperature-sensing neuron regulates thermotaxis behavior in C. elegans
Source: PLoS One. 2022 Dec 6;17(12):e0278343. doi: 10.1371/journal.pone.0278343 (PMC9725164; doi:10.1371/journal.pone.0278343)
Supplement: Attachment — Submitted filename: cGMP_rebuttal_221025_IM_IA_IM.docx [file pone.0278343.s008.docx]

Oct 26, 2022

Dear Dr. Emily Chenette,

We greatly appreciate the constructive and helpful suggestions for the improvement on our previous manuscript submitted to PLOS Genetics (PGENETICS-D-22-00684). We revised our manuscript by addressing reviewer’s comments. Thanks to the reviewer’s suggestions, we indeed noticed several points we had previously overlooked and even reached a new hypothesis that could possibly explain the difference of AFD excitability according to cultivation temperature, a. k. a. single-cell memory. Figures S2B, S3B, S4 and 6 were newly added this time. Below are the point-to-point responses to the comments of each reviewer.

Reviewer #1: cGMP dynamics that underlie thermosensation in temperature-sensing neuron regulates thermotaxis behavior in C elegans

The overarching aim of this study is to elucidate mechanisms by which temperature information is transduced through cellular signaling mechanisms to animal behavior. As a model system the authors utilize the AFD neuron of C. elegans and a FRET-based cGMP sensor, as well as calcium sensors. The authors then make use of C. elegans genetics to examine, primarily, the roles of GCYs and phosphodiesterases (PDE-1,2,3,5) with these sensors, and behavior, under different cultivation temperatures.

The reported work extends previous studies, primarily by examining the dynamics of cGMP in mutant backgrounds for GCYs and phosphodiesterases (PDE-1,2,3,5). It is unclear, however, how this FRET-based sensor performs as compared to other published cGMP sensors that were previously used in the same neuron and under similar conditions. The original findings and contributions of the study, above what was previously understood, have value in the context of the mutant backgrounds examined, but are modest as compared to published work (Inada et al., 2006; Takeishi et al., 2016; Woldermariam et al. 2019; Wang et al. 2013).

Major comments:

• Regarding figures 1a,b: As the authors point out, previous work has exemplified cGMP dynamics in AFD in response to thermal ramps (Woldermariam et al. 2019). The pattern of responses across the two fluorescent reporters look distinct, and at this point it is difficult to discern if that is an effect of the sensor or temperature stimulus. A useful control would be attempting the same training and test conditions as Woldermariam et al. figure 3 and comparing the results of this FRET-based sensor to the GFP-based sensor of the other work.

As the reviewer #1 pointed out, those two probes for cGMP, FlincG and cGI-500, can be possibly more precisely compared with the same training and test conditions as Woldermariam et al. figure 3 to clarify whether any difference exists between the reactivities of those two probes. Nevertheless, we independently reached conclusions that include and are consistent with conclusions by Wolermariam et al. by using cGI-500: 1. cGMP increases upon warming, 2. Onset temperature of cGMP increase is dependent on the past cultivation temperature, 3. cGMP dynamics is observed specifically in the sensory ending but not in soma.

• Regarding figure 1c: Previous work (e.g. Huang et al. 2020) have shown major disruptions to sensory tip structure in gcy-8,18,23 mutant animals. Do the authors see similar structural defects? If so, how do the authors decouple the effects on imaging caused by 1) structural loss of sensory tip and 2) loss of cGMP signaling from GCYs? Huang et al. observed similar structural defects in tax-2 or tax-4 mutant animals, which are presumably downstream from gcy signaling. A useful control might be testing cGMP dynamics at the sensory tip in tax-2 or tax-4 mutant animals, to decouple the effects of structural and signaling defects present in the GCY triple mutant animals.

As the reviewer pointed out, *gcy-8 gcy-18 gcy-23* mutants always show abnormal morphology of AFD sensory ending lacking microvilli as described by Huang et al. 2020. We imaged from these abnormal sensory endings with defective morphology. Imaging in *tax-4* mutant would be indeed useful to decouple the effects of the structural abnormality and the loss of cGMP synthesis in the *gcy-8 gcy-18 gcy-23* mutants as the reviewer pointed out. We actually transferred *njEx1285[gcy-8p::CGI-500]* array to *tax-4* mutants, but only found the probe totally invisible. There might be some feedback from Ca2+ influx through the CNG channels to transcription from the *gcy-8* promoter. These discussions were included in our manuscript.

• Regarding figure 3: Animals defective at sensing temperature seem to evenly spread across the gradient (e.g. gcy triple mutants in the bottom right of figure 2a). In contrast, the PDE mutants appear to remain near the center of the gradient, which could be equally well explained by a motility deficit. If the authors run wild type and PDE mutant animals in an arena absent of a thermal gradient, will PDE animals still display a movement deficit?

The analysis of locomotion without thermal gradient suggested by the reviewer can clarify whether the defect shown in Figure 3 is caused by the motility deficit or not. Nevertheless, locomotion of all the PDE mutants seemed normal on NGM plates, which was newly mentioned in our manuscript.

• Regarding figure 4: The disconnect between cGMP signaling and AFD calcium responses is perhaps the most surprising finding of the paper. The authors posit that a higher baseline cGMP level in PDE-5 mutant animals may move the signal out of the cGI-500 sensor’s operating range. A technical issue like this would be consistent with the rest of the data – PDE-5 mutant animals have similar thermotactic deficits as PDE-1,2 mutant animals, which display wild type cGMP and calcium responses (fig 5b-c). This hypothesis seems testable with the data already in hand by comparing the ratiometric values (rather than the delta R/R values) for each condition – do PDE-5 animals have a higher baseline cGI-500 signal compared to all of the other PDE mutants tested? How do other sensors perform (related to point 1)?

As suggested by the reviewer, we analyzed the cGMP baselines (R_0_) (S4 Fig C). R_0_ in *pde-5* did not clearly exceed that of wild type*.* We are not sure yet whether R_0_ properly reflect the basal cGMP level since R_0_ was not decreased in *gcy-23 gcy-8 gcy-18* triple mutants, where cGMP is supposed to be greatly decreased. We have not tried any other probes for cGMP so far, given that a FRET probe like cGI-500 is ratio-metric and cancels out difference in expression level of the probe in different strains if any.

• Regarding figure 6: Based on the authors hypothesis from figure 4 that PDE-5 mutants exhibit a higher baseline cGMP level, do the authors see an elevated cGMP baseline level in PDE-1,5 mutants?

Baseline analysis was also performed for *pde-5 pde-1* double mutant. Significant elevation of the baseline was also not detected in *pde-5 pde-1* (S4 Fig C)*.*

Minor comments:

• Please revise the following sentence at lines 139-142 for clarity: “These results suggest that the enzymatic activity of GCY-23 and GCY-8 is turned on at lower temperature than that of GCY-18, rather than that the downstream processes such as provision of cGMP from each GCY to CNG channels are temperature dependent, contribution of which is not excluded.”

The corresponding sentence was modified to clarify the ambiguity.

• Regarding figure 2b: The data for gcy-8,18 and gcy-23,18 animals appears considerably more noisy than wild type. Is there a change in baseline fluorescence, or some other effect, that would drive this change?

Increased noise in *gcy-8 gyc-18* and *gcy-23 gcy-18* double mutants was probably due to low fluorescence intensity (S4 Fig A). Therefore, GCY-18 might positively regulate expression from *gcy-8* promoter. R_0_ was increased in these double mutants, suggesting that GCY-23 and GCY-8 are aberrantly activated when existing alone. This was further discussed in our manuscript.

• Regarding figure 4a: The 17 degree cultivated PDE-5 mutants are not significantly different from their rescues – it is perhaps an overstatement to say ‘AFD-specific pde-5 expression rescued abnormality in thermotaxis of pde-5 mutants cultivated at both 17 deg C…’

We thank the reviewer for finding out the overstatement. The corresponding statement was modified,

• In the grids of line plots (cGMP/Ca++ dynamics with temperatures), it may help readability to space out the plots and add y-axis labels next to each column (not necessary to have them next to each plot), rather than just on the ends.

Plots were rearranged as suggested to improve readability.

• Figure 5 should be moved to the supplement. In figure 3 the authors introduce PDEs 1 and 5 having the strongest effects, while PDEs 2 and 3 have very minor (if any) effects. Focusing on PDEs 1 and 5 (figures 4 and 6) flow nicely, while the data in figure 5 are relatively uncompelling – the effect sizes of the mutants are quite small, the rescues are partial, and there is a disconnect between the behavior data and the cGMP/Ca++ signaling data.

We agree to the reviewer’s suggestion. Former Figure 5 was moved to the supplement.

Reviewer #2: This paper investigated how AFD encodes information of ambient temperature into neural activities by characterizing cGMP dynamics in AFD in response to temperature changing. They found cGMP level in AFD cilium can be increased by warming and decreased by cooling in a manner dependent on cultivation temperature. They found the cGMP dynamics can partially reflect AFD Ca2+ dynamics and worm thermotaxis behavior. They further showed that AFD-specific GCYs determine the onset temperature for cGMP increase via checking GCY-deficient mutants. Moreover, they described the contributions of phosphodiesterases (PDEs) to proper thermotaxis. Overall, the authors did a nice job in uncovering cGMP dynamics in AFD neuron. I have a few comments that I hope the authors will be able to address before it is considered for publication.

1. There is not a whole lot of information provided about the discrepancy among the abolished cGMP dynamics, the normal calcium changes, and the defective thermotaxis behavior. The authors should discuss the potential synergistic mechanisms (like pde-1 and pde-5) apart from pde-5 involvement alone.

cGMP dynamics in *pde-5 pde-1* double mutants might be different from that in *pde-5*, although it seemed totally abolished in both strains during our measurements possibly due to the limited sensitivity of the probe. We added this discussion in the manuscript.

2. The authors showed that AFD-specific expression of pde-1 or pde-2 partially rescued pde-1; pde-2 double mutants. A similar phenomenon happened in pde-5; pde-1 double mutants. Can simultaneous AFD expression of two PDEs fully rescue the defective behavioral and imaging phenotype to the level comparable with wild type?

Given that both PDE-1 and PDE-2 act in AFD, we expect the expression of both PDE-1 and PDE-2 will probably fully rescue the defective thermotaxis of *pde-1; pde-2,* although the difference is already very subtle and not always reliably detected between N2 and *pde-1* or *pde-2* single mutants and between N2 and *pde-1; pde-2* rescue strains expressing either PDE-1 or PDE-2 (S5 Fig A (Former Figure 5)). We did not observe any significant differences in both Ca2+ and cGMP dynamics even in *pde-1; pde-2* double mutants in our experimental condition (S5Fig B-D). We slightly modified the manuscript to better describe the experimental results.

Given that both PDE-1 and PDE-5 act in AFD, we expect the expression of both PDE-1 and PDE-5 will probably fully rescue the defective thermotaxis of *pde-5 pde-1*. We expect the defective cGMP dynamics in *pde-5 pde-1* (Figure 5B) will be rescued by expression of PDE-5 alone but not by PDE-1, since *pde-1* mutants did not show any noticeable defect in cGMP dynamics (S5 Fig B) and the defective cGMP in *pde-5* mutants were rescued by PDE-5 expression in AFD (Figure 4B). The defective Ca2+ dynamics in *pde-5 pde-1* (Figure 5C&D) will be probably restored by expression of either PDE-1 or PDE-5 alone since both *pde-1* and *pde-5* single mutants showed normal Ca2+ dynamics. The manuscript was very slightly modified.

3. The authors use different ramping rates for Figure 1 and Figure 2B (1° C/6sec and 1°C/20sec, respectively). Notably, for wild type animals, there is a prominent heat-induced cGMP response in accordance with the first round of heating-cooling regardless of previous cultivation temperatures. However, this initial response was not observed at slower ramping rate (as shown in Figure 2B). This should be pointed out and discussed.

We're not sure If we understand this comment correctly. We used slower ramping rate in Figure 2B to quantitatively compare the onset of cGMP increment, and started the thermal cycle from cooling because we knew the cGMP increment was more clearly observed during warming after cooling and because we wanted to reduce the recording time. Therefore, in Figure 2B, wild type animals’ cGMP dynamics starts from decrement, which were not so clear in three of *gcy* double mutants.

4. In terms of AFD soma, the dynamics of calcium response to heat is similar to that of sensory endings, but the cGMP curve is totally disrupted compared to cGMP dynamics of sensory endings. A more detailed analysis would be of interest.

Given the specific localization of GCYs and CNG channels and the cGMP and Ca2+ dynamics we described in this study, we expect cGMP synthesis activates CNG channels at the AFD sensory ending, and Ca2+ signaling is somehow propagated to soma. Since EGL-19 voltage-gated Ca2+ channel was shown to propagate Ca2+ signaling from sensory ending to soma in ASEL chemosensory neuron (Shindou 2019), similar mechanism might also function in AFD. We slightly elaborated on this point in our manuscript.

5. Based on Figure 2B and S2A, the responding thresholds of GCY double mutants were different from that of wild type. Detailed information about threshold change would be warranted (for example, by figuring out the exact threshold value of each mutant).

In addition to the quantification of threshold for cGMP dynamics shown in Figure 2C&D, we quantified threshold of Ca2 dynamics shown in former Figure S2 (current Figure S2 and S3).

During the analysis, we noticed that all the three *gcy* double mutants cultivated at 17°C showed prolonged Ca^2+^ signal at soma (S3 Fig, left). This suggests that the full set of GCYs is necessary for the Ca^2+^ shutdown. We mentioned this in the ‘Discussion’ part of our manuscript.

6. For gcy-23; gcy-8; gcy-18 triple mutant, why are the cGMP dynamics much more impaired (Figure 1C), but the thermotaxis behavioral phenotype is less defective than gcy-8; gcy-18 double mutant?

*gcy-23 gcy-8 gcy-18* triple mutant is totally insensitive to temperature (Kobayashi 2016) and therefore spreads evenly on a thermal gradient (Inada 2006, Figure 2A). On the other hand, *gcy-8 gcy-18* mutants cultivated at 23°C can still sense temperature but in an abnormal way, where cGMP and Ca2+ increases in response to warming from much lower temperature than their cultivation temperature as if the animals were cultivated at lower temperature. This mis-representation of temperature information, derived from mis-processing of either past cultivation temperature or current temperature, probably drive the animals toward the cold regions of thermal gradient.

This discussion was added to the corresponding part of the Result section.

7. Figure 1C showed that in GCYs triple mutant worms, cGMP dynamics in AFD cilium anti-correlated with warming and cooling, which is opposite to wt worms. They attribute this result to the effect of temperature change on the probe or the focus. I think they'd better do a negative control to give a more convincing reason. For example, they can record the cGMP dynamics in a temperature insensitive neuron by the same protocol.

We agree with the reviewer’s suggestion that an observation of cGMP dynamics in a temperature insensitive neuron under the same temperature program will better explain the fluorescence ratio change of cGI-500 seen in *gcy* triple mutants (Figure 1C). Nevertheless, we are certain that the increment and decrement of the fluorescence ratio change in response to warming and cooling seen in wild type reflects change of cGMP concentration.

8. Figure 2B showed that in GCY double mutant worms, especially in GCY-8/GCY18 and GCY-23/GCY-18 double mutant worms, cGMP dynamics in AFD cilium exhibited lower shrethold to warming (become more sensitive to warming). What is the possible reason

Figure 2B indicates that GCY-23 and GCY-8 get activated at low temperature compared to GCY-18 when existing alone. In contrast, in wild type animals that have both GCY-23 and GCY-8 cGMP increase is probably suppressed below the threshold. It will be probably the case also in *gcy-18* single mutant since it shows normal thermotaxis (Figure 2A) and almost normal Ca2+ dynamics (Wasserman et al., 2011). Therefore, co-existence of both GCY-23 and GCY-8 seems to suppress the activity of each other below the threshold. We further discussed on this point in our manuscript.

Reviewer #3: There has been significant progress in the genetic understanding of C. elegans thermotaxis thanks to the Sengupta, Mori, Goodman labs and others. The AFD thermosensory neuron has emerged as the major regulator of thermotaxis, responsible for both sensing temperature gradients to guide navigation and remembering the long-term memory of past temperatures to create a thermotactic set-point. A major advance in elucidating behavioral mechanisms was discovering that a set of guanylyl cyclase molecules (GCY-8, GCY-18 and GCY-23) and cyclic nucleotide gated channels (TAX-2 and TAX-4) are required for AFD-dependent thermotaxis behaviors. So far, AFD physiology has been largely limited to imaging intracellular calcium dynamics. New cGMP probes now permit direct measurement of this important signaling molecule. Here, such a probe is leveraged to assessing mutant phenotypes among the guanylyl cyclase and phosphodiesterase molecules that regulate cGMP levels in AFD.

This is a very nice study that systematically analyzes many of the known molecules that regulate AFD thermosensation, analyzed with a new cGMP sensor. The authors confirm the requirement of guanylyl cyclases in thermotaxis, and confirm and extend measurements of temperature-dependent changes in cGMP levels that had been detected using another sensor by Woldermariam et al. 2019. The new results are honestly and thoroughly presented. This study goes a long way towards reconciling and supporting results from the many different labs who work on C. elegans thermotaxis.

My only suggestion is that the key results are presented in Figure 2 – measurements of cGMP levels in double mutants that are raised at one cultivation temperature (T=23). I suggest performing the cGMP imaging experiments across the same set of conditions for behavioral analyses (see Figure 2A), which includes single mutants and animals raised at a different cultivation temperature. The paper is already nice and thorough, but these additional experiments would strengthen their claims and further illuminate any differences in the relative contributions of the three guanylyl cyclase molecules at different temperatures and different thermotactic set-points.

This is an important paper for the field, but would be improved with additional straightforward imaging experiments that would make them fully

complementary to their more comprehensive behavioral experiments.

We thank reviewer #3 for acknowledging the importance of the systematic analysis of behavior and Ca2+- and cGMP dynamics in AFD in mutants for cGMP-related genes. We agree to the reviewer’s suggestion that our manuscript will be more complete with the dataset of behavior and imaging for the strains with all the possible combinations of *gcy* mutations. However, we expect that cGMP dynamics of single *gcy* mutants will be mostly the same as wild type given their mostly intact thermotaxis behavior (Figure 2A).

-----------------------------------------------------------------------------------------------------

Again, we would like to thank the reviewers and the editors of PLOS Genetics for their many helpful comments and are happy that we had the opportunity to improve our manuscript. We hope that the manuscript will now be worthy of consideration for publication in PLOS ONE.

Sincerely yours,

Ikue Mori, Ph.D.

Neuroscience Institute, Graduate School of Science,

Nagoya University

Furo-cho, Chikusa-ku

Nagoya 464-8602, Japan

TEL +81-52-789-4560 (or -4558)

FAX +81-52-789-4558

e-mail: m46920a@nucc.cc.nagoya-u.ac.jp
