# Supplementary material for: cGMP dynamics that underlies thermosensation in temperature-sensing neuron regulates thermotaxis behavior in C. elegans
Source: PLoS One. 2022 Dec 6;17(12):e0278343. doi: 10.1371/journal.pone.0278343 (PMC9725164; doi:10.1371/journal.pone.0278343)
Supplement: Peer review history

# Author comment: Author response to previous submission

**DOI:** 10.1371/journal.pone.0278343.r001

**Transfer Alert**

This paper was transferred from another journal. As a result, its full editorial history (including decision letters, peer reviews and author responses) may not be present.

27 Oct 2022

Attachment Submitted filename: cGMP_rebuttal_221025_IM_IA_IM.docx

# Editor report: Decision Letter 0

**DOI:** 10.1371/journal.pone.0278343.r002

**Authors:** Erik A. Lundquist

15 Nov 2022

cGMP dynamics that underlies thermosensation in temperature-sensing neuron regulates thermotaxis behavior in C. elegans

PONE-D-22-29567

Dear Dr. Mori,

We’re pleased to inform you that your manuscript has been judged scientifically suitable for publication and will be formally accepted for publication once it meets all outstanding technical requirements.

Within one week, you’ll receive an e-mail detailing the required amendments. When these have been addressed, you’ll receive a formal acceptance letter and your manuscript will be scheduled for publication.

An invoice for payment will follow shortly after the formal acceptance. To ensure an efficient process, please log into Editorial Manager at http://www.editorialmanager.com/pone/, click the 'Update My Information' link at the top of the page, and double check that your user information is up-to-date. If you have any billing related questions, please contact our Author Billing department directly at authorbilling@plos.org.

If your institution or institutions have a press office, please notify them about your upcoming paper to help maximize its impact. If they’ll be preparing press materials, please inform our press team as soon as possible -- no later than 48 hours after receiving the formal acceptance. Your manuscript will remain under strict press embargo until 2 pm Eastern Time on the date of publication. For more information, please contact onepress@plos.org.

Kind regards,

Erik A. Lundquist

Academic Editor

PLOS ONE

Journal requirements:

When submitting your revision, we need you to address these additional requirements.

1. Thank you for stating the following in the Acknowledgments Section of your manuscript: 

"We thank H. Matsuyama for critical reading; and K. Ikegami, Y. Murakami, for technical assistance. Some strains were provided by the Caenorhabditis Genetics Center (CGC), which is funded by NIH Office of Research Infrastructure Programs (P40 OD010440). This work was supported by JSPS KAKENHI Grant Numbers JP 16H01272, JP 16H02516 and JP 18H04693 (to IM)."

We note that you have provided funding information that is not currently declared in your Funding Statement. However, funding information should not appear in the Acknowledgments section or other areas of your manuscript. We will only publish funding information present in the Funding Statement section of the online submission form. 

Please remove any funding-related text from the manuscript and let us know how you would like to update your Funding Statement. Currently, your Funding Statement reads as follows: 

"This study was funded by following grants to I.M.: Ministry of Education, Culture, Sports, Science and Technology (MEXT) KAKENHI JP16H01272 and JP18H04693 (https://www.mext.go.jp/en/policy/science_technology/researchpromotion/title01/detail01/1374077.htm), and Japan Society for the Promotion of Science (JSPS) Grants-in-Aid for Scientific Research JP16H02516 (https://www.jsps.go.jp/english/e-grants/index.html). The funders had no role in study design, data collection and analysis, decision to publish, or preparation of the manuscript."

Please include your amended statements within your cover letter; we will change the online submission form on your behalf.

# Editor report: Acceptance letter

**DOI:** 10.1371/journal.pone.0278343.r003

**Authors:** Erik A. Lundquist

22 Nov 2022

PONE-D-22-29567 

cGMP dynamics that underlies thermosensation in temperature-sensing neuron regulates thermotaxis behavior in C. elegans 

Dear Dr. Mori:

I'm pleased to inform you that your manuscript has been deemed suitable for publication in PLOS ONE. Congratulations! Your manuscript is now with our production department. 

If your institution or institutions have a press office, please let them know about your upcoming paper now to help maximize its impact. If they'll be preparing press materials, please inform our press team within the next 48 hours. Your manuscript will remain under strict press embargo until 2 pm Eastern Time on the date of publication. For more information please contact onepress@plos.org.

If we can help with anything else, please email us at plosone@plos.org. 

Thank you for submitting your work to PLOS ONE and supporting open access. 

Kind regards, 

PLOS ONE Editorial Office Staff

on behalf of

Dr. Erik A. Lundquist 

Academic Editor

PLOS ONE
